# Repurposed Medicines for Viruses With Epidemic or Pandemic Potential: A Horizon Scan

**DOI:** 10.1002/prp2.70271

**Published:** 2026-05-18

**Authors:** Sola Akinbolade, Rhiannon Potter, Alex Inskip, Jane Nesworthy, Kirsti Brock, Gill Norman

**Affiliations:** ^1^ National Institute for Health and Care Research (NIHR) Innovation Observatory, Population Health Sciences Institute, Faculty of Medical Sciences Newcastle University Newcastle UK

**Keywords:** direct‐acting antivirals, drug repositioning, drug repurposing, emerging viral infections, epidemic preparedness, horizon scanning, host‐targeted therapies, pandemic preparedness, viruses

## Abstract

Viruses such as Ebola, Marburg, influenza, mpox, MERS‐CoV, SARS‐CoV, and SARS‐CoV‐2 may be considered pathogens of epidemic or pandemic concern. Developing novel antiviral medicines can be time‐consuming and resource intensive. Repurposing existing medicines with known or potential antiviral activity offers a faster, cost‐effective strategy to expand treatment options during public health emergencies. This scan aimed to map current investigational activity involving repurposed medicines for these viruses. A horizon scanning approach was employed, starting with a targeted search in Embase followed by a systematic search of ClinicalTrials.gov to identify developmental stages of relevant technologies. Eligible technologies included UK‐ or EU‐licensed medicines being investigated for antiviral use, while vaccines, unlicensed medicines, and treatments already approved for the target viruses were excluded. From the literature, 196 repurposed technologies were identified, and the expanded search on the clinical trials registry revealed 58 technologies in active clinical development. Interventional trial activity was limited to influenza and SARS‐CoV‐2, with 29 technologies for SARS‐CoV‐2 and two influenza technologies advancing to phase III evaluation. For other viruses, candidate repurposed technologies were identified only at preclinical or exploratory stages. Frequently investigated pharmacological classes included direct‐acting antivirals, immunomodulators, and anti‐inflammatory agents. While repurposing represents a potentially rapid strategy for therapeutic deployment, inclusion in this horizon scan does not imply clinical efficacy. Rigorous preclinical validation, pharmacokinetic feasibility assessment, and mechanistic confirmation remain essential before clinical translation.

## 1 Introduction

Viruses are microscopic organisms capable of infecting living hosts. While not all viruses are contagious, those that are capable of transmission can spread rapidly within human populations. Some viruses are considered potential epidemic or pandemic threats because of their high transmissibility and ability to cause significant illness and/or death [1]. A virus cannot replicate outside of living cells of a host organism; however, once it infiltrates a host cell, it uses components within the host cell to multiply, often killing the host cell and causing damage to the host organism [2]. Viruses contain either an RNA (ribonucleic acid) or DNA (deoxyribonucleic acid) genome surrounded by a virus‐coded protein coat [2]. Genetic changes or mutations in these genetic materials can result in new functions or enhanced characteristics of the virus, potentially enhancing their ability to infect hosts, cause disease, or evade immune responses. Such mutations may lead to the emergence of new viral strains or variants [1].

Epidemic‐ or pandemic‐prone viruses are typically characterized by their high transmissibility and ability to cause severe disease. They can arise from either zoonotic (animal‐to‐human) or non‐zoonotic (human‐to‐human or environmental) transmission pathways. Zoonotic viruses such as Ebola and Marburg exemplify the risk associated with cross‐species transmission from wildlife to humans [3]. These RNA filoviruses can cause severe hemorrhagic fever and are transmitted through direct contact with infected bodily fluids, with outbreaks sustained by subsequent human‐to‐human transmission [3]. Respiratory viruses, including influenza and the coronaviruses MERS‐CoV (Middle East respiratory syndrome coronavirus), SARS‐CoV (severe acute respiratory syndrome coronavirus), and SARS‐CoV‐2, represent another major pandemic threat due to their potential for airborne transmission and rapid human‐to‐human spread [3, 4]. Influenza viruses cause annual global epidemics of seasonal infections and can give rise to pandemics when new strains emerge against which the population has little or no pre‐existing immunity [4]. Members of the Orthopoxvirus genus, such as smallpox and mpox (formerly known as monkeypox), further illustrate the diversity of viral threats [5, 6]. Mpox can be transmitted zoonotically, typically through bites or scratches from infected animals, but human‐to‐human transmission can also occur through close contact [6]. Notably, smallpox was eradicated in the 1970s through a global vaccination programme, representing a landmark achievement in public health [7].

Historically, viral pandemics have caused high mortality and continue to pose a global threat due to emerging and re‐emerging outbreaks, driven by the evolution of viral variants [3]. These challenges highlight the critical importance of having effective therapeutic options readily available for future epidemic or pandemic responses. Promising strategies for combating viral infections often involve direct‐acting antivirals which target specific components of the virus, or host‐targeted therapies that modulate the host immune response to inhibit viral replication and minimize immune‐related damage [8].

The discovery and development of novel medicines often face significant challenges, including long timelines, high costs, and the risk of unforeseen adverse events. These are further compounded by complex regulatory hurdles, which can increase expenses and delay the introduction of new therapies to market [9, 10]. Existing antiviral medicines originally developed and approved for specific infections can be evaluated for their efficacy and effectiveness against other viral threats. This strategy, known as medicine repurposing or repositioning, involves identifying new therapeutic uses for already approved medicines outside the scope of their original indication [9, 10]. Repurposed medicines often have established safety profiles, which can reduce the associated risk of novel drug development [9]. There is potentially a reduction in both development time and cost, largely due to being able to use existing data from completed preclinical studies, safety assessments, and, in some cases, formulation development [10]. As a result, repurposing efforts can often begin from proof‐of‐concept phase II clinical trials, with the primary objectives of evaluating efficacy, as well as assessing safety and tolerability [11]. The reduction in development time and cost associated with repurposing can be particularly valuable in a viral pandemic where immediate treatment options are crucial for controlling the spread and severity of the disease. Repurposing existing direct‐acting antivirals or host‐targeted agents could offer a potential pathway for developing effective treatments against various viral diseases.

This scan aims to identify and map recent investigational activity involving repurposed medicines with known or potential antiviral activity against viruses of epidemic or pandemic concern, including Ebola, Marburg, influenza, mpox, MERS‐CoV, SARS‐CoV, and SARS‐CoV‐2. Additionally, it seeks to offer insights to support future antiviral repurposing efforts.

## 2 Method

A horizon scanning approach was employed to identify antiviral medicines currently in development or under investigation for potential repurposing. This strategy involved systematically searching bibliographic and clinical trials databases to capture emerging innovations and potential therapeutic opportunities within the antiviral field [12].

The scan for antiviral medicines encompassed those traditionally classified as ‘conventional’ or direct‐acting antivirals as well as medicines from other therapeutic classes that possess known or potential antiviral activity, which may be repurposed for the treatment of emerging or re‐emerging priority viruses. The inclusion of a medicinal product in this horizon scan reflects its identification in published literature or clinical trial registries and does not constitute an assessment of antiviral potency, mechanistic validity, pharmacokinetic feasibility, or clinical effectiveness.

### 2.1 Eligibility Criteria

To ensure a systematic and transparent approach, predefined inclusion/exclusion criteria were established to determine the eligibility of studies, as follows in (Table 1).

**TABLE 1:** Eligibility criteria.

Criterion	Inclusion	Exclusion
Population	Interventional studies involving one or more of the following viruses: Ebola. Marburg. Influenza. Mpox (monkeypox). MERS‐CoV. SARS‐CoV. SARS‐CoV‐2.	Studies not involving the specified viruses
Intervention	Repurposed direct‐acting antivirals or medicines from other therapeutic classes with known or potential antiviral activity evaluated for the treatment of one or more of the viral diseases of interest. Medicines licensed in the UK or EU. Monotherapies and combination therapies.	Vaccines (prophylactic or therapeutic). Medical devices, diagnostics, or digital tools. Dietary supplements or herbal remedies. Medicines unlicensed in the UK or EU. Medicines already licensed for the same viral disease under investigation, unless being evaluated in combination with another licensed product not yet approved that indication.
Horizon	English‐language articles published from 2022 onwards for Ebola, Marburg, influenza, and mpox, and from 2024 onwards for MERS‐CoV, SARS‐CoV, and SARS‐CoV‐2. All types of published studies were eligible. Interventional clinical trials (phases I‐IV) registered on ClinicalTrials.gov with a primary completion date from January 2022 onwards, across the targeted viral diseases.	Articles published in languages other than English. Articles published outside the defined time window. Clinical trials with a primary completion date prior to January 2022. Observational or non‐interventional studies.
Data source	Bibliographic database (Embase). Clinical trials registry (ClinicalTrials.gov).	

### 2.2 Search Strategy

#### 2.2.1 Literature Search

A literature search was conducted in April 2025 using the Embase [13] bibliographic database to identify recent publications relevant to the development of repurposed antiviral medicines. The search was limited to articles published from 2022 onwards for Ebola, Marburg, influenza, and mpox. For MERS‐CoV, SARS‐CoV, and SARS‐CoV‐2, the search was restricted to publications from 2024 onwards to focus on more refined, post‐pandemic strategic research and to limit the volume of data.

The search strategy was developed by an Information Specialist (AI) and incorporated a comprehensive set of keywords, including terms such as “antiviral agent,” “antiviral therapy,” “antiviral activity,” “drug repurposing,” “drug repositioning,” “off‐label drug use,” “licensed,” “MHRA‐approved,” “EU‐approved,” “UK‐approved,” as well as disease‐specific terms and synonyms for each target virus. The full search strategy, including all search strings and filters, is provided in Appendix A.

All identified articles were exported into Microsoft Excel for manual screening against the predefined inclusion criteria, followed by extraction of eligible medicinal products. This process ensured a systematic and consistent identification of relevant medicinal products.

#### 2.2.2 Clinical Trials Search

Following the identification of technologies from the literature search, their known aliases and alternative names were systematically used to search the ClinicalTrials.gov [14] registry for interventional studies, accounting for variability in how products may be reported. This approach ensured comprehensive capture of their developmental stages.

To further broaden the search scope, additional searches were conducted without specifying product names, focusing instead on trial records containing the term “antiviral” (or “anti‐viral”) in the title or summary. This multi‐layered strategy maximized the identification of relevant antiviral medicinal products while maintaining a manageable volume of results.

To focus on the most recent therapeutic developments, only trials with a primary completion date from January 2022 onwards were included. All eligible records were exported to Microsoft Excel for manual screening and further assessment.

### 2.3 Study Screening and Selection

All identified studies were independently screened in Microsoft Excel against the predefined eligibility criteria by members of the review team (S.A., R.P., J.N., or K.B.). Each study was assessed and marked as either “include” or “exclude” based on its relevance to the study objectives. If a screener encountered uncertainty or had a query about a particular study, it was flagged for further review. These flagged studies were then assessed by a second reviewer, and any discrepancies were resolved through discussion to reach consensus on inclusion or exclusion. This screening process aimed to enhance consistency, reduce bias, and ensure rigorous selection of relevant studies.

### 2.4 Data Extraction

Data were manually extracted from all included studies to generate an overview of each identified antiviral medicine, detailing the medicine name (whether investigated as a monotherapy or in combination) and its corresponding targeted viral disease. Extraction was conducted using a standardized Microsoft Excel data extraction form to ensure consistency across reviewers. Any uncertainties or discrepancies encountered during the extraction process were documented and resolved through discussion within the full review team. Upon completion of data extraction, a quality assurance check was performed by a second reviewer to ensure accuracy and consistency of the extracted information.

### 2.5 Verification of Repurposing Status

To verify regulatory approval status of each identified antiviral medicine, the UK and EU licensing information was manually cross‐referenced using the electronic medicines compendium [15] or European Medicines Agency [16] websites respectively. This step ensured that all shortlisted medicines were already approved for use in at least one indication by either the UK or EU regulatory authorities and that unlicensed medicines were excluded. Additionally, this verification process helped identify whether each medicine had already been licensed for the treatment of any of the targeted viral diseases—Ebola, Marburg, influenza, mpox, MERS, SARS, or COVID‐19. Medicines already licensed for a specific viral disease were excluded if they were being evaluated for the same viral disease indication. However, an exception was made for medicines already licensed for a specific viral disease if they were being evaluated in combination with another licensed medicine that had not yet been approved for that viral indication. This approach allowed the review to capture novel combination therapies with potential to advance antiviral treatment options beyond current standards of care.

## 3 Results

The literature search yielded 274 results. Following thorough screening against the predefined eligibility criteria, 112 articles were included in the analysis, from which 196 unique technologies were extracted. The identified medicinal products were classified as “technologies,” defined as either a single medicine or a combination of medicines investigated for the treatment of a specific viral disease. The technologies identified represent reported investigational activity rather than confirmed antiviral efficacy and varied substantially in the strength of supporting mechanistic and pharmacological evidence.

The repurposed technologies represented a spectrum of development stages, including preclinical evaluation, active clinical investigation, or theoretical candidates proposed through computational or mechanistic studies. However, distinctions between these stages were not systematically captured during data screening or extraction. To identify technologies in active clinical development, an additional search of the ClinicalTrials.gov registry was conducted as described in the Methods section.

**FIGURE 1:**
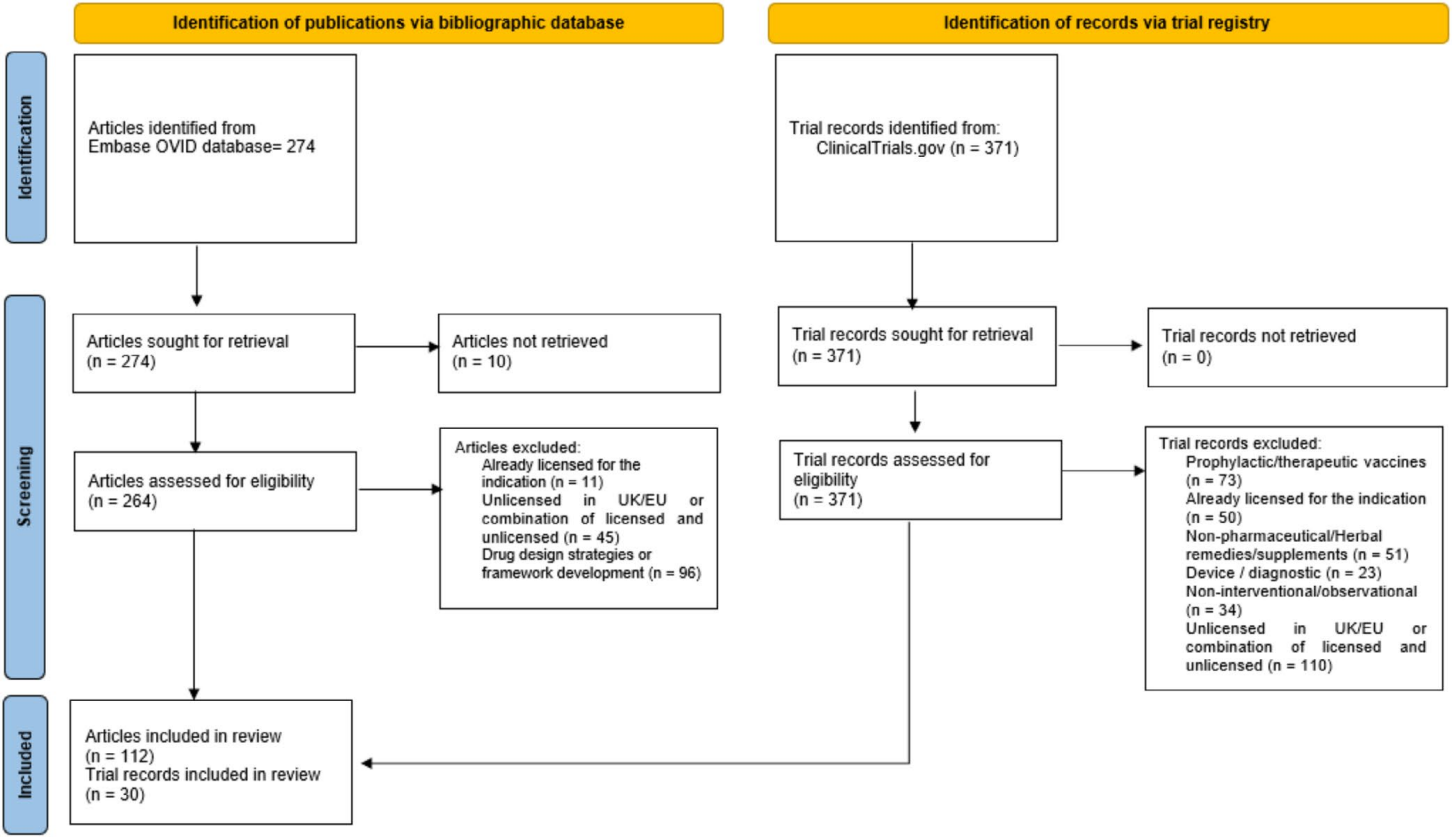
Flow diagram illustrating the selection process and the final number of included articles and clinical trial records.

This registry search yielded 371 records, of which 30 clinical trials met the predefined eligibility criteria. These 30 trials collectively yielded 58 technologies, as several trials investigated multiple therapeutic interventions across different treatment arms, reflecting the diversity of treatment strategies being explored. Figure 1 presents the flow diagram [17] illustrating the process of study identification, screening, and inclusion.

**FIGURE 2:**
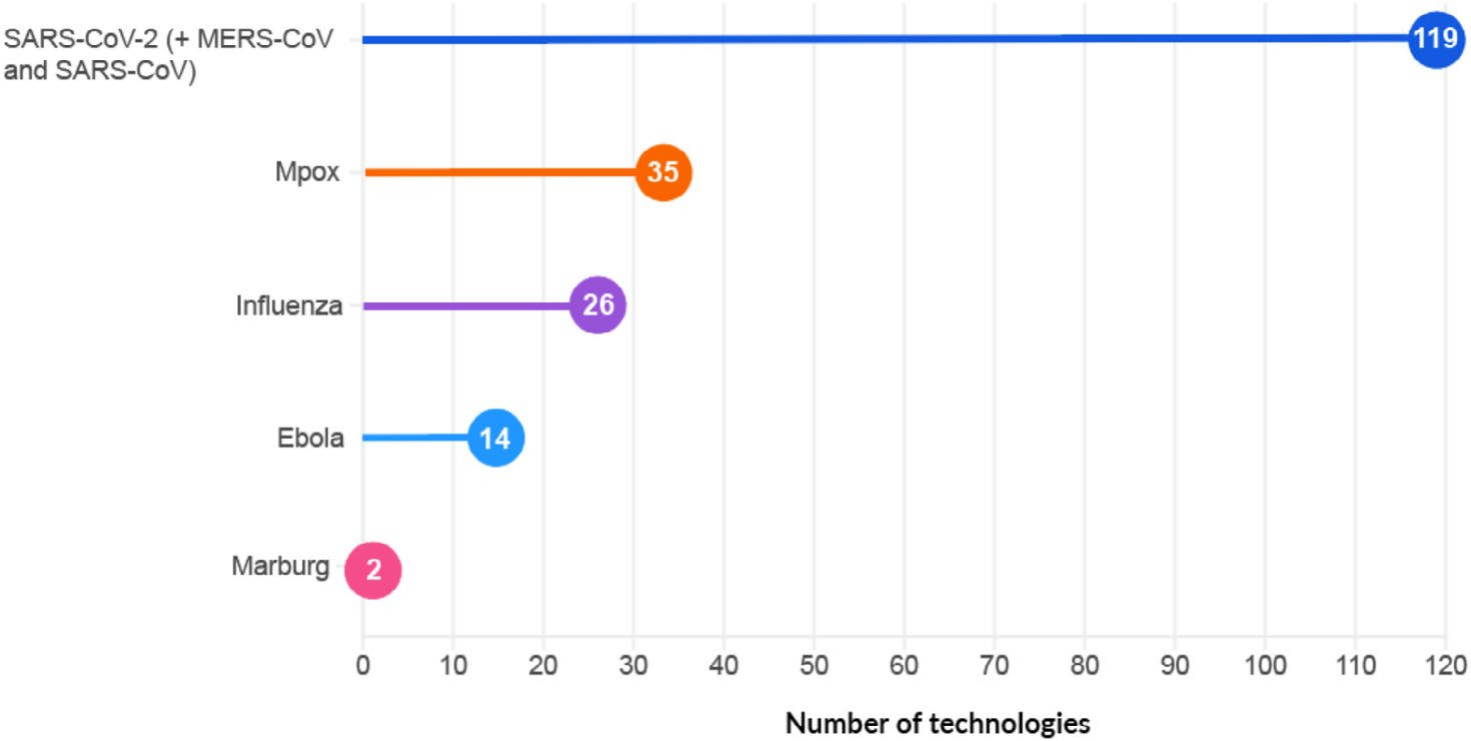
Overview of identified technologies from published literature, encompassing those in preclinical evaluation, ongoing clinical investigation, or proposed as potential candidates for repurposing.

The repurposed technologies identified from the published literature are grouped according to their target viruses (Figure 2) and are described in detail in the following sections.

### 3.1 Ebola

The analysis of published literature identified 14 technologies proposed for repurposing as potential treatments for Ebola virus disease (EVD). Among them, only three were direct‐acting antivirals; the rest included diverse classes such as selective estrogen receptor modulators, antibiotics, and thrombopoietin receptor agonists. Notably, two of these repurposed candidates were originally approved as cancer therapies (see Table S1).

**FIGURE 3:**
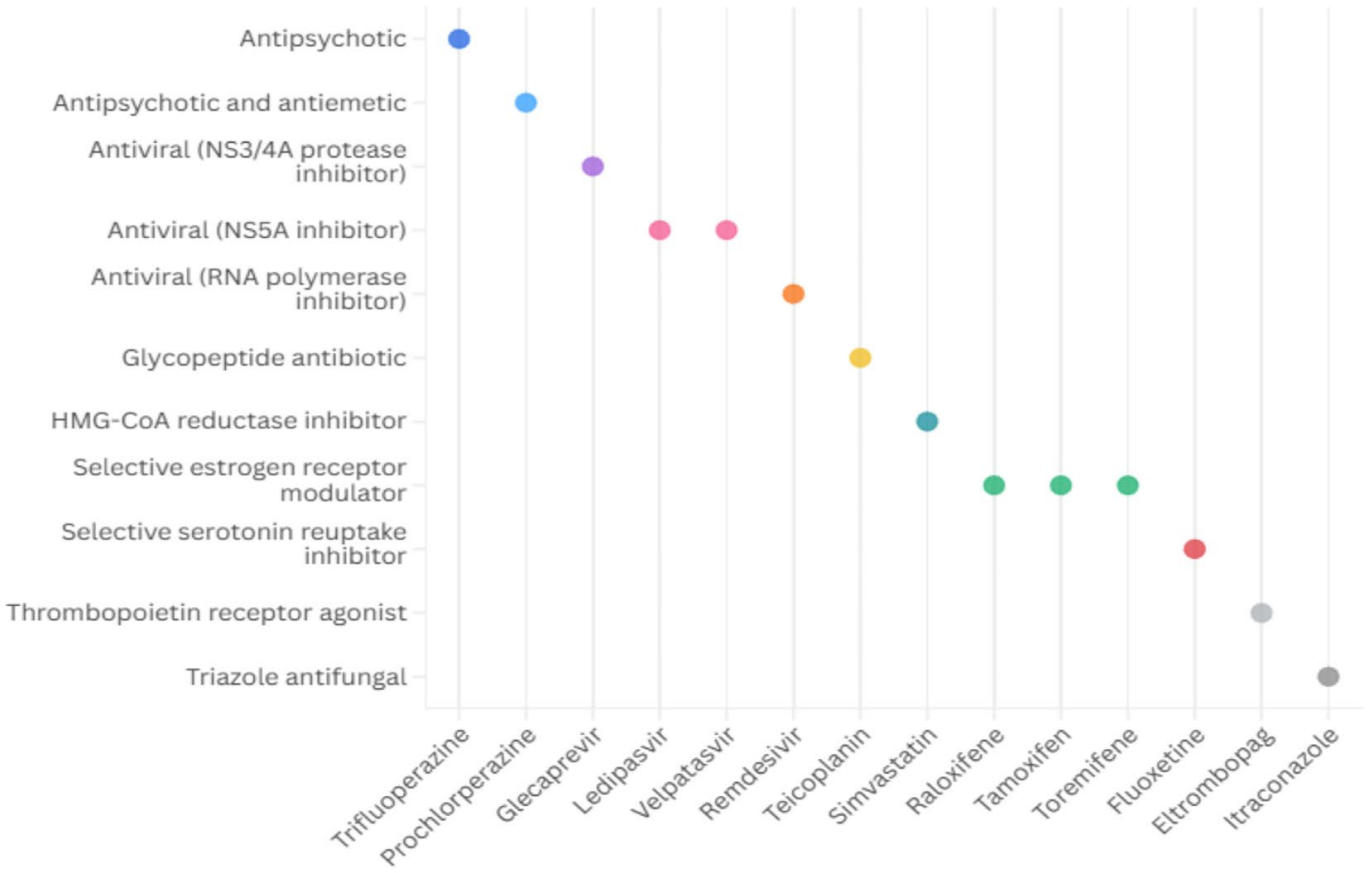
Therapeutic classes of repurposed medicines evaluated for the treatment of Ebola.

EVD is characterized by the virus's ability to target and destroy immune cells, leading to immune suppression and the onset of severe disease [18]. The progression and outcome of EVD depend largely on the host's ability to suppress viral replication and the virus's ability to evade or manipulate the immune response [18]. As such, treatment strategies identified in literature for EVD focused on approaches such as the use of direct‐acting antivirals targeting the virus itself (e.g., glecaprevir, velpatasvir), agents that block viral entry or replication (e.g., selective estrogen receptor modulators [tamoxifen, raloxifene]), therapies that modulate host immune responses (e.g., selective serotonin reuptake inhibitors [fluoxetine]), or statins for their anti‐inflammatory effect and vascular protection (e.g., simvastatin) (Figure 3) (full details available in Table S1).

### 3.2 Marburg

According to the World Health Organization, there are currently no approved antiviral therapies for the treatment of Marburg virus disease, and potential candidates remain under investigation [19]. In the absence of licensed therapeutic options, current development efforts appear to prioritize the evaluation of existing antivirals for potential repurposing. Our analysis identified two candidates under investigation for Marburg virus disease, namely remdesivir, an RNA polymerase inhibitor originally approved for the treatment of COVID‐19, and bictegravir, an integrase strand transfer inhibitor used in the management of human immunodeficiency virus infection. Both medicines have demonstrated antiviral activity in their original indications, supporting their exploration as potential therapies against Marburg virus disease (full details available in Table S2).

### 3.3 Influenza

Antiviral medicines approved for the treatment of influenza have demonstrated the ability to reduce symptom severity and lower the risk of complications when administered early in the course of infection [20]. Ongoing research is increasingly focused on developing therapeutic options that may effectively combat the virus while mitigating the emergence of drug‐resistant viral strains [21].

**FIGURE 4:**
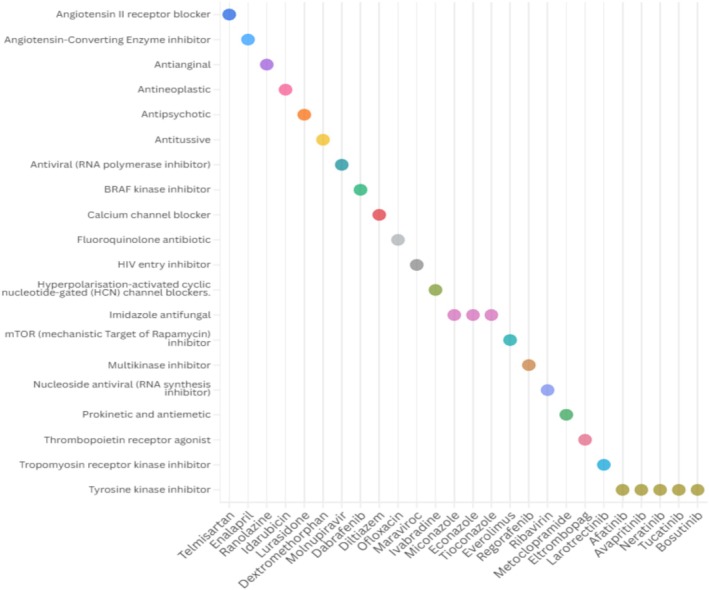
Therapeutic classes of repurposed medicines evaluated for the treatment of influenza.

Our analysis identified 26 technologies currently being investigated for potential repurposing in the treatment of influenza virus infection. These candidates represent a broad spectrum of therapeutic classes, including direct‐acting antivirals (e.g., molnupiravir); imidazole antifungals (e.g., econazole) with potential host‐directed antiviral effects; thrombopoietin receptor agonists (e.g., eltrombopag) aimed at enhancing platelet production; tyrosine kinase inhibitors (e.g., afatinib) that may interfere with viral entry and replication; and antineoplastic agents (e.g., idarubicin) with immunomodulatory properties (Figure 4) (full details available in Table S3). All identified candidates were originally developed and approved for indications unrelated to influenza, encompassing both cancer and non‐cancer conditions, highlighting the potential of cross‐indication repurposing strategies.

### 3.4 Mpox

Thirty‐five technologies were identified in published literature as candidates for repurposing in the treatment of mpox virus. Among these, two were combination therapies involving tecovirimat (currently the only approved antiviral for mpox) [22, 23] paired with medicines licensed for non‐mpox indications. The first combination included fenofibrate, a lipid‐lowering agent approved for the treatment of hypertriglyceridaemia and mixed hyperlipidaemia, administered alongside tecovirimat. The second involved mycophenolate, an immunosuppressive agent approved for the prophylaxis of acute transplant rejection, also combined with tecovirimat.

**FIGURE 5:**
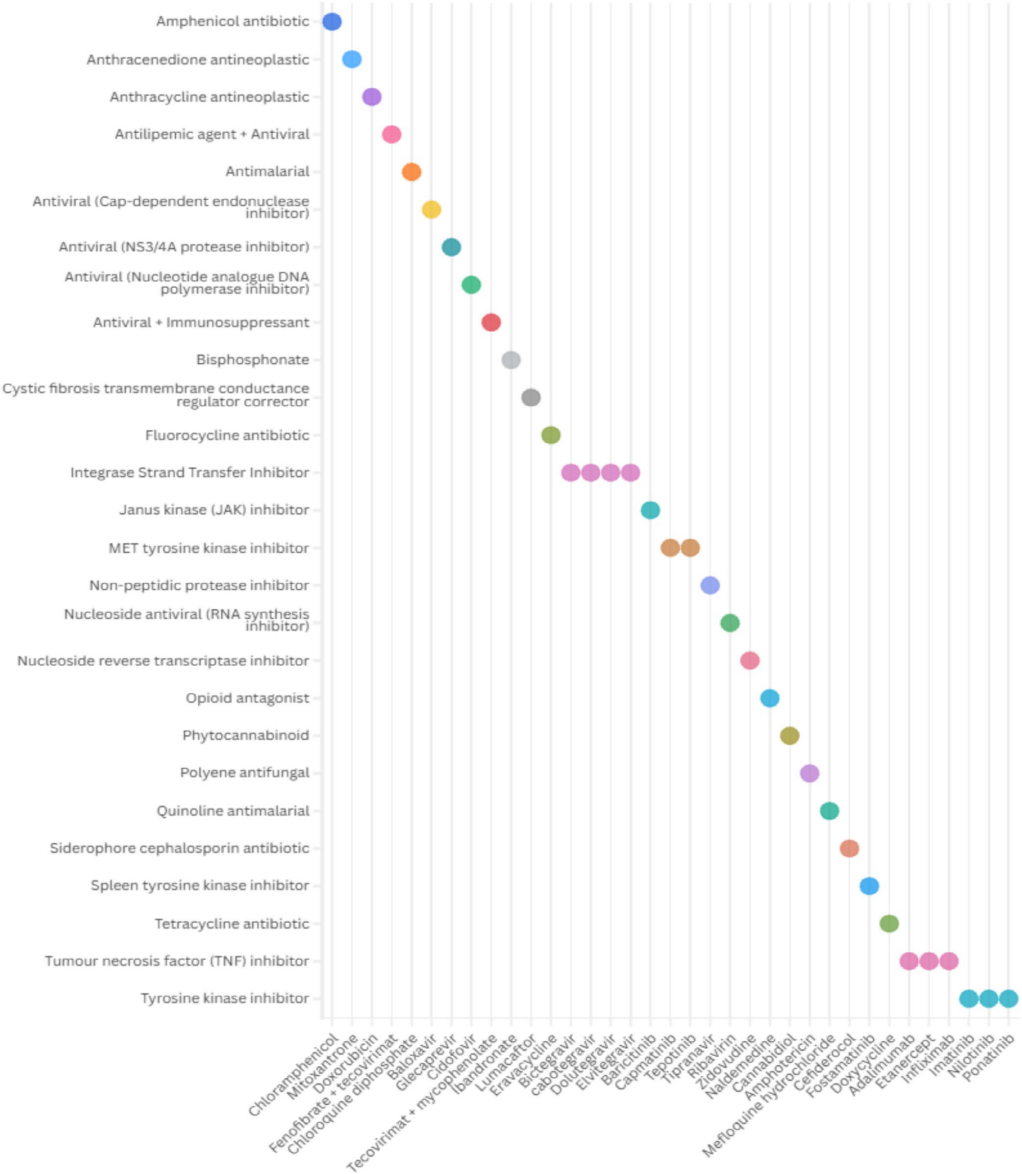
Therapeutic classes of repurposed medicines evaluated for the treatment of mpox.

The remaining identified technologies were monotherapies exploring the repurposing potential of diverse pharmacological classes. These included antivirals (e.g., baloxavir) aimed at directly inhibiting viral replication; integrase strand transfer inhibitors (e.g., cabotegravir) investigated for their potential host‐modulatory effects; tumor necrosis factor inhibitors (e.g., adalimumab) used for their immunosuppressive properties to mitigate inflammation; and tyrosine kinase inhibitors (e.g., ponatinib) which may disrupt viral entry and replication (Figure 5) (full details available in Table S4). This breadth of therapeutic classes reflects a growing interest in host‐targeted and multi‐mechanism approaches for the treatment of mpox.

### 3.5 MERS‐CoV, SARS‐CoV, and SARS‐CoV‐2

Middle East respiratory syndrome coronavirus (MERS‐CoV), severe acute respiratory syndrome coronavirus (SARS‐CoV), and SARS‐CoV‐2 are closely related RNA viruses responsible for outbreaks of severe respiratory disease with high case fatality rates. These coronaviruses exhibit considerable overlap in clinical presentation, including fever, cough, and respiratory distress, making them clinically difficult to distinguish based on symptoms alone [24].

**FIGURE 6:**
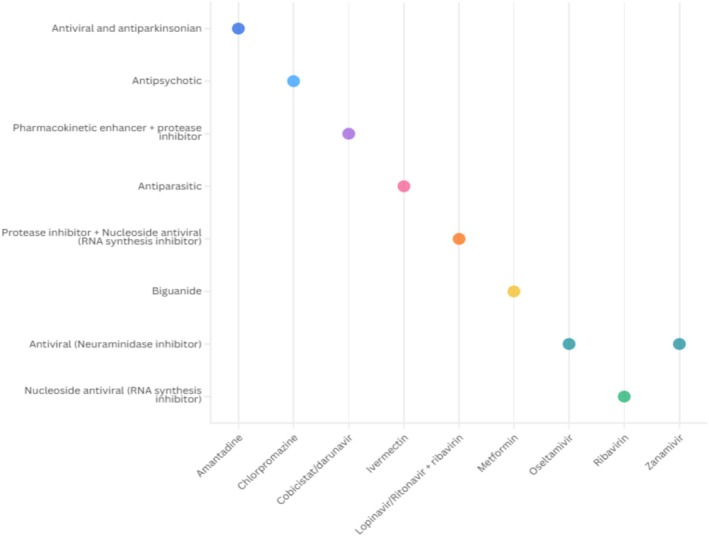
Therapeutic classes of repurposed medicines evaluated for the treatment of MERS‐CoV, SARS‐CoV, and SARS‐CoV‐2.

A total of 119 technologies were identified from literature as potential repurposing options for the treatment of coronavirus infections. Of these, only nine were proposed as candidates for use across MERS‐CoV, SARS‐CoV, and SARS‐CoV‐2 (Figure 6), while the remainder were exclusively evaluated for SARS‐CoV‐2 (full details available in Table S5). These technologies encompassed a wide range of pharmacological classes, including direct‐acting antivirals such as zanamivir and ledipasvir, which target viral replication pathways. Antimalarial agents like chloroquine diphosphate and the combination of pyronaridine with artesunate were explored for their potential antiviral and immunomodulatory properties. Tyrosine kinase inhibitors, including entrectinib and nilotinib, were investigated for their ability to disrupt viral entry and replication. Selective serotonin reuptake inhibitors such as fluoxetine and fluvoxamine were evaluated for their immunomodulatory effects, while alkylating agents like carboplatin and cisplatin were considered for their cytotoxic activities (Figure 6).

## 4 Repurposed Technologies in Active Clinical Development

Following the identification of technologies from the literature, a search of ClinicalTrials.gov was conducted to determine their developmental status. The search revealed that, among the viruses of interest, only SARS‐CoV‐2 and influenza are currently the focus of active interventional trials evaluating repurposed antiviral medicines.

Our analysis identified 30 unique interventional clinical trials, from which 58 technologies were retrieved (see Tables S6 and S7). The discrepancy between the number of technologies and trials reflects the inclusion of multiple treatment arms within individual trials, encompassing both monotherapy and combination therapy approaches. In several instances, the investigated therapies involved combinations of medicines, where at least one was already approved for the target viral disease, and the other was repurposed from a non‐related indication. These combinations represent novel therapeutic strategies aimed at enhancing efficacy or addressing resistance mechanisms.

**FIGURE 7:**
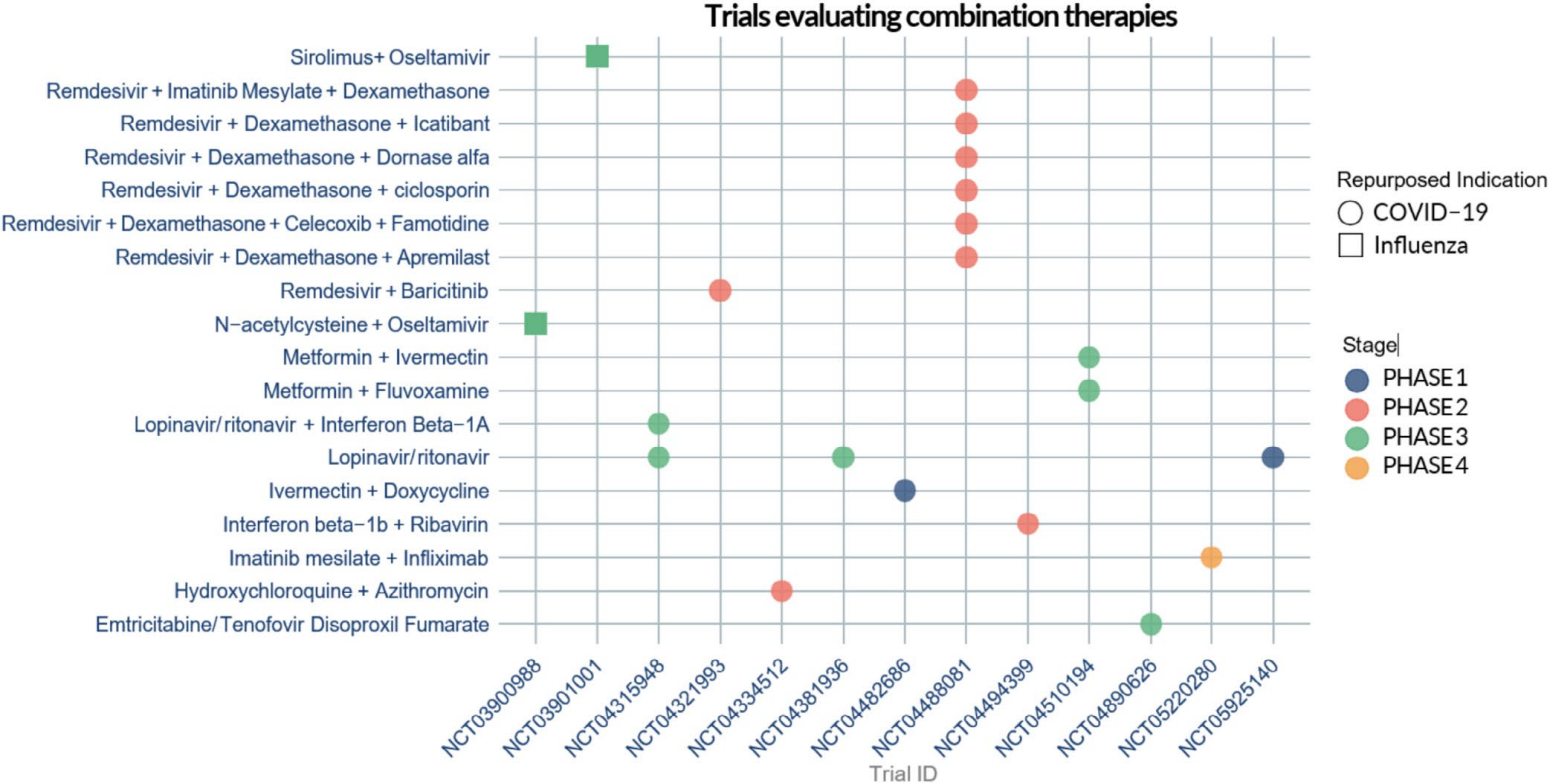
Graph showing the repurposed combination therapies in clinical development to treat SARS‐CoV‐2 (COVID‐19) and influenza.

Among the technologies identified, 18 were repurposed combination therapies, reflecting an interest in leveraging synergistic mechanisms of action to enhance antiviral efficacy or improve clinical outcomes. Only two of these combinations were being investigated for the treatment of influenza. The first combined oseltamivir, a neuraminidase inhibitor approved for influenza, with sirolimus, an mTOR (mammalian target of rapamycin) inhibitor typically used as an immunosuppressant; this strategy combines direct antiviral activity with modulation of host immune pathways. The second combination therapy paired oseltamivir with N‐acetylcysteine, a mucolytic and antioxidant agent with potential to reduce oxidative stress and inflammation, thereby complementing antiviral activity. Both influenza‐focused combination therapies were in phase III clinical development, indicating they are at an advanced stage of evaluation (Figure 7) (full details available in Table S6).

In contrast, combination therapies under investigation for SARS‐CoV‐2 were more diverse. The regimens included direct‐acting antivirals (e.g., remdesivir), corticosteroids (e.g., dexamethasone), and immunomodulators such as Janus kinase inhibitors (e.g., baricitinib), among others. Of these SARS‐CoV‐2‐focused combination therapies, six were in phase III clinical development, indicating substantial progress towards potential clinical adoption (Figure 7) (full details available in Table S7). This diversity of treatment strategies underscores the complex pathophysiology of SARS‐CoV‐2 and highlights the importance of multidimensional approaches that can address both viral and host‐mediated drivers of disease.

**FIGURE 8:**
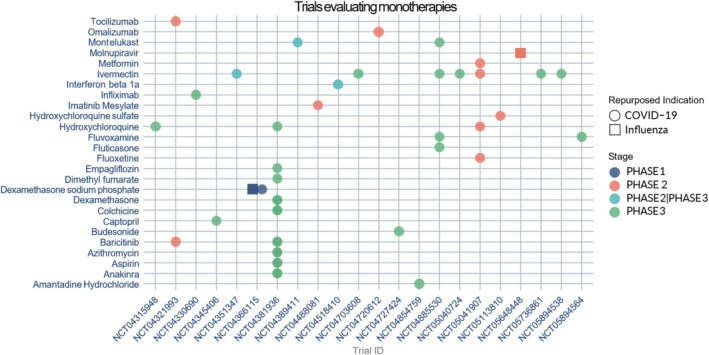
Graph showing the repurposed monotherapies in clinical development to treat SARS‐CoV‐2 (COVID‐19) and influenza.

A total of 40 repurposed monotherapies were identified across the included clinical trial records, with some, such as ivermectin and hydroxychloroquine, being evaluated in multiple studies. While a broad range of monotherapy candidates is under investigation for SARS‐CoV‐2, the pipeline for influenza appears far more limited. Only two repurposed medicines were identified as being in development for influenza: molnupiravir, a direct‐acting antiviral currently in phase II clinical development, and dexamethasone sodium phosphate, a corticosteroid undergoing phase I evaluation (Figure 8) (full details available in Table S6).

For SARS‐CoV‐2, the monotherapies encompassed a diverse range of pharmacological classes, reflecting strategies aimed at inhibiting viral replication and modulating host immune responses. These included interleukin‐6 (IL‐6) receptor inhibitors (e.g., tocilizumab), monoclonal antibodies (e.g., omalizumab), biguanides (e.g., metformin), and leukotriene receptor antagonists (e.g., montelukast), among others. Importantly, 23 of these monotherapies are in phase III clinical development, indicating substantial progress towards potential clinical adoption (Figure 8) (full details available in Table S7). This diversity of mechanisms highlights the multifactorial nature of SARS‐CoV‐2 pathogenesis and the need for a broad therapeutic approach to address various stages of infection, mitigate complications, and improve patient outcomes.

Of the 30 trials identified, only five had reported results, all investigating treatments for SARS‐CoV‐2 (COVID‐19); however, none demonstrated a clinically meaningful benefit in patients. In NCT04518410, interferon beta‐1a and other interventions assessed in the trial showed no clear efficacy. Similarly, NCT04510194, which evaluated metformin, ivermectin, and fluvoxamine as monotherapies and in combination, found that none of the treatments prevented hypoxaemia, emergency department visits, hospitalization, or death. Ivermectin was further evaluated as a monotherapy at different doses in two additional trials. In NCT05736861, ivermectin at 400 μg/kg conferred no benefit in time to sustained recovery or in hospitalization and death rates. Likewise, NCT05894538, which tested ivermectin at a higher dose of 600 μg/kg, showed no difference in recovery time or hospitalization rates compared with placebo. Finally, NCT05894564, evaluating fluvoxamine 100 mg twice daily, demonstrated no improvement in time to sustained recovery, hospitalization, or death outcomes. Other trials identified through the scan remain ongoing. Results from these studies have not yet been reported and are expected to provide further evidence on the repurposing of licensed medicines for the treatment of SARS‐CoV‐2 and influenza.

## 5 Discussion

This work provides a comprehensive horizon scan of medicines reported as potential candidates for repurposing or currently in clinical development for a defined set of viruses with epidemic or pandemic potential. By synthesizing evidence from published literature and registered clinical trials, we identified a diverse landscape of investigational repurposing activity, which may represent novel uses for medicines already licensed in the UK or EU. The identified technologies included medicines traditionally classified as antivirals as well as medicines from other therapeutic classes that may possess known or potential antiviral activity. Some technologies involved combining medicines already licensed for a specific viral disease with others not yet approved for that indication, creating novel therapeutic strategies. The inclusion of a medicine in this horizon scan, however, reflects its identification in published literature or clinical trial registries and does not constitute an assessment of antiviral potency, mechanistic validity, pharmacokinetic feasibility, or clinical effectiveness.

Clinical trial activity was observed to be concentrated exclusively on SARS‐CoV‐2 and influenza, with no active interventional trials identified for repurposed antiviral medicines targeting Ebola, Marburg, mpox, MERS‐CoV, or SARS‐CoV. The excluded trials for these viral diseases primarily reflected efforts to develop novel (unlicensed) therapeutics, a stronger emphasis on preventive interventions, or further evaluations of treatments already approved for these viral diseases. Although innovation through new drug development is essential, repurposing existing licensed medicines offers a pragmatic and potentially faster route to expand therapeutic options in the face of global health concerns.

The greatest concentration of repurposing activity was observed for SARS‐CoV‐2, reflecting the unprecedented global research mobilization following the COVID‐19 pandemic. A wide array of pharmacological classes was identified, targeting different aspects of SARS‐CoV‐2 pathogenesis, including direct‐acting antivirals, host‐modulatory agents such as interleukin‐6 inhibitors, and immunomodulators. The range of mechanisms observed highlights the multifactorial nature of SARS‐CoV‐2 infection and the need for broad therapeutic strategies capable of addressing viral replication, inflammation, and downstream complications. A total of 29 repurposed technologies, encompassing both monotherapies and combination regimens, had progressed to phase III clinical evaluation, representing significant advancement towards potential clinical adoption and strengthening preparedness for future pandemic responses. In addition, 18 technologies were undergoing phase II evaluation, highlighting a robust mid‐stage development pipeline with the potential to further expand therapeutic options. Therapeutic exploration for SARS and MERS was underrepresented; although historically significant, these viruses have received less recent research attention, likely reflecting a shift in focus towards SARS‐CoV‐2 as the more immediate coronavirus threat.

For influenza, a long‐standing seasonal infection, the identified technologies targeted either direct viral inhibition or modulation of the host immune response. Only two repurposed monotherapies were in active clinical development for influenza: molnupiravir, a direct‐acting antiviral in phase II trials, and dexamethasone, a corticosteroid undergoing phase I evaluation. In addition, two novel combination therapies involving oseltamivir (currently approved for influenza) were identified. Both combination therapies were in phase III clinical development, indicating substantial progress towards potential adoption into clinical practice.

For mpox, we identified 35 technologies from literature; two involved combination therapies involving tecovirimat (currently the only medicine approved for mpox) paired with other medicines licensed for other indications other than mpox. The exclusive approval of tecovirimat underscores the need for alternative options, especially in cases of resistance or treatment failure. The diversity of mechanisms among candidate therapies reflects growing interest in host‐targeted approaches as complementary strategies. No clinical trials for mpox met the inclusion criteria, as they either focused on prophylactic vaccines, unlicensed medicines, or further evaluations of tecovirimat, which is already approved for mpox.

Therapeutic development for Ebola and Marburg viruses remains limited. For Ebola, 14 technologies were identified, while only two were reported for Marburg, all of which were extracted from literature. No clinical trials met the inclusion criteria for either virus, as existing studies primarily focused on prophylactic vaccines or involved unlicensed medicines.

A key feature of this scan was its focus on UK‐ or EU‐licensed medicines with potential for repositioning. By excluding unlicensed medicines, the findings map investigational repurposing activity among established products and may highlight opportunities for the strategic reuse of existing medicines. However, inclusion in this scan does not imply demonstrated antiviral efficacy, pharmacokinetic feasibility, or clinical validity, all of which require rigorous preclinical and clinical evaluation. The COVID‐19 pandemic underscored the risks of advancing products into clinical trials based solely on in vitro activity in non‐physiologically relevant models. Effective antiviral repurposing must be grounded in well‐characterized viral or host targets, robust validation in relevant preclinical models, and pharmacokinetic/pharmacodynamic evidence demonstrating achievable tissue exposure relative to antiviral potency (e.g., EC90 thresholds). Such insights may nevertheless inform the design of adaptive trial platforms and support strategic decision‐making during public health emergencies. Although none of the trials with reported results demonstrated clinical benefit, others remain ongoing, and their findings may yet clarify the feasibility and limitations of repurposing licensed medicines.

Despite its strengths, this scan has limitations. We did not perform a formal appraisal of mechanistic robustness, pharmacokinetic feasibility, or translational likelihood of identified candidates. Consequently, the presence of a technology within this scan should not be interpreted as evidence of clinical promise. The exclusion of non‐English articles and reliance on a single literature database and clinical trials registry may have omitted relevant studies. The emphasis on recent publications (from 2022 onwards) helped capture timely innovations but may have missed earlier repurposing efforts still relevant to current development. Additionally, the analysis focused on medicines with existing UK or EU licenses, which may not reflect the full global pipeline of repurposing candidates.

## 6 Implications and Future Directions

The findings of this scan highlight the potential of repurposing licensed medicines for emerging and re‐emerging viral threats. Repurposing existing direct‐acting antivirals or other pharmacological classes with potential antiviral activities may offer a potentially rapid, cost‐effective means of expanding treatment options in future outbreaks, provided that sufficient preclinical and clinical data support their use.

Indirect‐acting antiviral medicines or host‐targeted therapies, which modulate host pathways essential for viral replication or pathogenesis, represent a promising strategy to overcome challenges related to viral mutation and drug resistance. These medicines may offer broad‐spectrum activity and sustained efficacy across different viral variants. Repurposing medicines could significantly expand treatment options during the early stages of a viral outbreak, when virus‐specific therapies are unavailable or still in development.

Looking ahead, collaboration among researchers, research funders, pharmaceutical industry, policymakers, public health agencies, and regulatory bodies will be essential to improve pandemic preparedness. Such coordination is also vital for accelerating the regulatory evaluation and adoption of repurposed medicines, ensuring that safe and effective therapeutic options can be rapidly mobilized in future public health emergencies.

## Author Contributions

S.A. drafted the manuscript. A.I. conducted the literature and clinical trial searches. S.A., R.P., J.N., and K.B. carried out data screening and extraction. S.A. and R.P. analyzed the data. G.N. reviewed and provided feedback on the final draft of the manuscript. All authors provided critical feedback and helped refine the final version.

## Funding

This project is funded by the NIHR (HSRIC‐2016‐10 009)/Innovation Observatory. The views expressed are those of the authors and not necessarily those of the NIHR or the Department of Health and Social Care.

## Conflicts of Interest

The authors declare no conflicts of interest.

## Supporting information


**Table S1:** Potential candidates for the treatment of Ebola.
**Table S2:** Potential candidates for the treatment of Marburg.
**Table S3:** Potential candidates for the treatment of Influenza.
**Table S4:** Potential candidates for the treatment of Monkeypox (mpox).
**Table S5:** Potential candidates for the treatment of MERS‐CoV, SARS‐CoV, SARS‐CoV‐2 (COVID‐19).
**Table S6:** Interventional clinical trials identified for influenza.
**Table S7:** Interventional clinical trials identified for SARS‐CoV‐2 (COVID‐19).

## Data Availability

All data supporting the findings of this study are provided in the Supporting Information.

## Appendix A Literature Search

### Search Conducted Using Embase Bibliographic Database on 8th April 2025

**Table:**

1	*monkeypox/	4161
2	*smallpox/	3175
3	*monkeypox virus/	1258
4	*smallpox virus/	519
5	(*orthopoxvirus/or *vaccinia virus/) and (monkeypox* or mpox* or m‐pox* or mpxv or smallpox*).mp.	1156
6	*poxvirus infection/and (monkeypox* or mpox* or m‐pox* or mpxv or smallpox*).mp.	197
7	(monkeypox* or mpox* or m‐pox* or mpxv or smallpox*).ti,kf.	9233
8	*ebola hemorrhagic fever/or *marburg hemorrhagic fever/	4838
9	*filovirus infection/and (ebola* or marburg*).mp.	143
10	*ebolavirus/or *bundibugyo ebolavirus/or *reston ebolavirus/or *zaire ebolavirus/or *marburgvirus/	3578
11	*filovirus/and (ebola* or marburg*).mp.	327
12	(ebola* or marburg*).ti,kf.	10 767
13	exp *Influenza/	60 639
14	exp *Influenza virus/	40 291
15	(influenza* or flu).ti,kf.	113 428
16	or/1–15	144 364
17	(drug? or inhibitor? or compound? or candidate? or small molecule? or therap* or antivir* or anti‐vir*).mp.	22 576 021
18	exp antivirus agent/or antiviral therapy/or exp. antiviral activity/or antiinfective agent/	1 885 591
19	([vaccine? or vaccinat*] not [drug? or inhibitor? or compound? or small molecule? or therap* or antivir* or anti‐vir*]).ti.	254 245
20	(17 or 18) not 19	22 507 798
21	16 and 20	64 070
22	Drug repositioning/	13 249
23	“off label drug use”	12 648
24	(licensed or FDA‐approved or MHRA‐approved or EU‐approved or UK‐approved or unlicensed or off‐label* or repurpos* or reposition*).ti,kf. or (licensed or FDA‐approved or MHRA‐approved or EU‐approved or UK‐approved or unlicensed or off‐label* or repurpos* or reposition*).ab./freq = 2	43 457
25	21 and (or/22–24)	300
26	Limit 25 to (English language and year = “2022–Current”)	112
27	*coronavirus disease 2019/or *asymptomatic coronavirus disease 2019/or *coronavirus infection/or *middle east respiratory syndrome/	342 549
28	*sars‐related coronavirus/or exp. *sars coronavirus/or exp. *severe acute respiratory syndrome coronavirus 2/or exp. *Middle East respiratory syndrome coronavirus	60 467
29	(sars* or severe acute respiratory syndrome? or mers* or middle east* respiratory syndrome? or coronavir* or covid or covid19).ti,kf.	461 683
30	or/27–29	475 459
31	30 and 20	202 176
32	31 and (or/22–24)	3478
33	32 and (antivir* or anti‐vir* or inhibit* or replication).mp.	2496
34	Limit 33 to (english language and year = “2024–Current”)	169
35	26 or 34	274

*Note:* https://ovidsp.ovid.com/ovidweb.cgi?T=JS&NEWS=N&PAGE=main&SHAREDSEARCHID=33aweiyOv88FokAu0t518E52voOXIJOodtWtOtFtfp31LQLjui7R6zBLTTBE08umy (login required).
