# Supplementary material for: Repurposed Medicines for Viruses With Epidemic or Pandemic Potential: A Horizon Scan
Source: Pharmacol Res Perspect. 2026 May 18;14(3):e70271. doi: 10.1002/prp2.70271 (PMC13182729; doi:10.1002/prp2.70271)
Supplement: Supplementary file 1 — Table S1: Potential candidates for the treatment of Ebola. Table S2: Potential candidates for the treatment of Marburg. Table S3: Potential candidates for the treatment of Influenza. Table S4: Potential candidates for the treatment of Monkeypox (mpox). Table S5: Potential candidates for the treatment of MERS‐CoV, SARS‐CoV, SARS‐CoV‐2 (COVID‐19). Table S6: Interventional clinical trials identified for influenza. Table S7: Interventional clinical trials identified for SARS‐CoV‐2 (COVID‐19). [file PRP2-14-e70271-s001.docx]

**Supplementary tables detailing the reported repurposed technologies (Tables 1- 7)**

**Supplementary Table 1 –** **Potential candidates for the treatment of Ebola**

| **Medicines** | **Therapeutic Class (A-Z)** | **UK/EU Approved Indication** | **Cancer Y/N** | **References** |
| --- | --- | --- | --- | --- |
| Trifluoperazine | Antipsychotic | Depressive symptoms secondary to anxiety, Agitation, Nausea and vomiting, Schizophrenia and in other psychoses, Severe psychomotor agitation and of dangerously impulsive behaviour | No | Almeida-Pinto 2024,^1^ Zeidan 2024^2^ |
| Prochlorperazine | Antipsychotic and antiemetic | Vertigo due to Ménière's disease, Labyrinthitis and other causes, Nausea and vomiting, Migraine | No | Almeida-Pinto 2024,^1^ Zeidan 2024^2^ |
| Glecaprevir | Antiviral (NS3/4A protease inhibitor) | Chronic hepatitis C | No | Broni 2023^3^ |
| Ledipasvir | Antiviral (NS5A inhibitor) | Chronic hepatitis C | No | Broni 2023^3^ |
| Velpatasvir | Antiviral (NS5A inhibitor) | Chronic hepatitis C | No | Broni 2023^3^ |
| Remdesivir | Antiviral (RNA polymerase inhibitor) | Covid-19 | No | Barghash 2024,^4^ Nascimento 2022^5^ |
| Teicoplanin | Glycopeptide antibiotic | Complicated skin and soft tissue infections, Bone and joint infections, Hospital acquired pneumonia, Community acquired pneumonia, Complicated urinary tract infections, Infective endocarditis, Peritonitis associated with continuous ambulatory peritoneal dialysis, Clostridium difficile infection | No | Barghash 2024,^4^ Espano 2024^6^ |
| Simvastatin | HMG-CoA reductase inhibitor | Hypercholesterolaemia, Cardiovascular prevention | No | Almedia-Pinto 2024^1^ |
| Raloxifene | Selective oestrogen receptor modulator | Osteoporosis | No | Almeida-Pinto 2024,^1^ Nascimento 2022^5^ |
| Tamoxifen | Selective oestrogen receptor modulator | Anovulatory infertility, Breast cancer | Yes | Almeida-Pinto 2024,^1^ Nascimento 2022^5^ |
| Toremifene | Selective oestrogen receptor modulator | Breast cancer | Yes | Almeida-Pinto 2024,^1^ Nascimento 2022^5^ |
| Fluoxetine | Selective serotonin reuptake inhibitor | Bulimia nervosa, Major depressive disorders, obsessive-compulsive disorder | No | Kummer 2022^7^ |
| Eltrombopag | Thrombopoietin receptor agonist | Acquired aplastic anaemia, Hepatitis C, Primary immune thrombocytopenia | No | Broni 2023^3^ |
| Itraconazole | Triazole antifungal | Aspergillosis, Candidosis, Cryptococcosis, Histoplasmosis | No | Almeida-Pinto 2024,^1^ Kummer 2022,^7^ Vanmechelen 2022^8^ |

**Supplementary Table 2 – Potential candidates for the treatment of Marburg**

| **Medicines** | **Therapeutic Class** | **UK/EU Approved Indications** | **Cancer Y/N** | **References** |
| --- | --- | --- | --- | --- |
| Remdesivir | Antiviral (RNA polymerase inhibitor) | COVID-19 | No | Martins 2025^9^ |
| Bictegravir | Integrase Strand Transfer Inhibitor | Human immunodeficiency virus | No | Singh 2025^10^ |

**Supplementary Table 3 – Potential candidates for the treatment of Influenza**

| **Medicines** | **Therapeutic Class (A-Z)** | **UK/EU Approved Indications** | **Cancer Y/N** | **References** |
| --- | --- | --- | --- | --- |
| Telmisartan | Angiotensin II receptor blocker | Hypertension, Cardiovascular prevention in manifest atherothrombotic cardiovascular disease or patients with type 2 diabetes mellitus with documented target organ damage | No | Taye 2023^11^ |
| Enalapril | Angiotensin-Converting Enzyme inhibitor | Heart failure, Hypertension, Cardiovascular event prevention | No | Ghimire 2022^12^ |
| Ranolazine | Antianginal | Add on treatment for stable angina pectoris | No | Taye 2024^11^ |
| Idarubicin | Antineoplastic | Acute non-lymphocytic leukaemia, Advanced breast cancer, Second line relapsed acute lymphoblastic leukaemia | Yes | Bordoloi 2023^13^ |
| Lurasidone | Antipsychotic | Schizophrenia | No | Mtambo 2022^14^ |
| Dextromethorphan | Antitussive | Cough | No | Xie 2022^15^ |
| Molnupiravir | Antiviral (RNA polymerase inhibitor) | COVID-19 | No | Padey 2024^16^ |
| Dabrafenib | BRAF kinase inhibitor | Melanoma | Yes | Meineke 2022^17^ |
| Diltiazem | Calcium channel blocker | Angina pectoris, Hypertension | No | Padey 2024^16^ |
| Ofloxacin | Fluoroquinolone antibiotic | Acute pyelonephritis and complicated urinary tract infections, non-gonococcal urethritis and cervicitis, Gonococcal urethritis and cervicitis due to susceptible *Neisseria gonorrhoeae*, Acute exacerbations of chronic obstructive pulmonary disease, Uncomplicated cystitis, Urethritis | No | Taye 2023^11^ |
| Maraviroc | HIV entry inhibitor | In combination with other medicinal products for human immunodeficiency virus | No | Xie 2022^15^ |
| Ivabradine | Hyperpolarisation-activated cyclic nucleotide-gated (HCN) channel blockers. | Chronic stable angina pectoris, Chronic heart failure | No | Ghimire 2022^12^ |
| Miconazole | Imidazole antifungal | Mycotic infections of the skin and superinfections due to Gram positive bacteria | No | Bordoloi 2023^13^ |
| Econazole | Imidazole antifungal | Candida/yeast infections, Vulvovaginitis and mycotic balanitis, Dermatophytosis and pityriasis versicolor | No | Bordoloi 2023^13^ |
| Tioconazole | Imidazole antifungal | Nail infections | No | Bordoloi 2023^13^ |
| Everolimus | mTOR (mechanistic Target of Rapamycin) inhibitor | Neuroendocrine tumours of pancreatic origin, Neuroendocrine tumours of gastrointestinal or lung origin, Renal cell carcinoma | Yes | Sun 2025^18^ |
| Regorafenib | Multikinase inhibitor | Colorectal cancer, Gastrointestinal stromal tumours, Hepatocellular carcinoma | Yes | Meineke 2022^17^ |
| Ribavirin | Nucleoside antiviral (RNA synthesis inhibitor) | In combination with other drugs: Chronic hepatitis c | No | Li 2023^19^ |
| Metoclopramide | Prokinetic and antiemetic | Nausea and vomiting | No | Taye 2023^11^ |
| Eltrombopag | Thrombopoietin receptor agonist | Primary immune thrombocytopenia, Hepatitis c, Acquired aplastic anaemia | No | Mtambo 2022^14^ |
| Larotrectinib | Tropomyosin receptor kinase inhibitor | Solid tumours | Yes | Meineke 2022^17^ |
| Afatinib | Tyrosine kinase inhibitor | Non-small cell lung cancer | Yes | Meineke 2022^17^ |
| Avapritinib | Tyrosine kinase inhibitor | Gastrointestinal stromal tumour, Systemic mastocytosis | Yes | Meineke 2022^17^ |
| Neratinib | Tyrosine kinase inhibitor | Breast cancer | Yes | Meineke 2022^17^ |
| Tucatinib | Tyrosine kinase inhibitor | Breast cancer | Yes | Mtambo 2022^14^ |
| Bosutinib | Tyrosine kinase inhibitor | Chronic myeloid leukaemia | Yes | Xie 2022^15^ |

**Supplementary Table 4 – Potential candidates for the treatment of Monkeypox (mpox)**

| **Medicines** | **Therapeutic Class (A-Z)** | **UK/EU Approved Indications** | **Cancer Y/N** | **References** |
| --- | --- | --- | --- | --- |
| Chloramphenicol | Amphenicol antibiotic | Bacterial conjunctivitis, Typhoid, Meningitis, Gram-negative and Gram-positive organisms | No | Podduturi 2024^20^ |
| Mitoxantrone | Anthracenedione antineoplastic | Breast cancer, non-Hodgkin’s lymphoma, Acute myeloid leukaemia | No | Preet 2022^21^ |
| Doxorubicin | Anthracycline antineoplastic | Breast cancer, Sarcoma, Small-cell carcinoma of the lung, Hodgkin disease or non-Hodgkin lymphoma | Yes | Sahoo 2023^22^ |
| Fenofibrate + tecovirimat | Antilipemic agent + Antiviral | Hypertriglyceridaemia, Mixed hyperlipidaemia \| Smallpox, Monkeypox, Cowpox | No | Vuorio 2022^23^ |
| Chloroquine diphosphate | Antimalarial | Active rheumatoid arthritis, Malaria, Amoebic hepatitis and abscess, Discoid and systemic lupus erythematosus | No | Horton 2025^24^ |
| Baloxavir | Antiviral (Cap-dependent endonuclease inhibitor) | Influenza | No | Hashemi 2024,^25^ Horton 2025,^24^ Rabaan 2024^26^ |
| Glecaprevir | Antiviral (NS3/4A protease inhibitor) | Chronic hepatitis C | No | Li 2023^27^ |
| Cidofovir | Antiviral (Nucleotide analogue DNA polymerase inhibitor) | Cytomegalovirus retinitis | No | Aldhaeefi 2023,^28^ Bhattacharjee 2024,^29^ Bojkova 2023,^30^ Borkotoky 2024,^31^ Ezat 2023,^32^ Horton 2025,^24^ Islam 2022,^33^ Lam 2022,^34^ Rejinold 2025,^35^ Shannon 2025,^36^ Vuorio 2022,^23^ Yousaf 2025^37^ |
| Tecovirimat + mycophenolate | Antiviral + Immunosuppressant | Smallpox, Monkeypox and Cowpox \| Prophylaxis of acute transplant rejection | No | Borkotoky 2024,^31^ Witwit 2025^38^ |
| Ibandronate | Bisphosphonate | Osteoporosis | No | Podduturi 2024^20^ |
| Lumacaftor | Cystic fibrosis transmembrane conductance regulator corrector | Cystic fibrosis | No | Dutt 2023,^39^ Khan 2024,^40^ Li 2023^27^ |
| Eravacycline | Fluorocycline antibiotic | Complicated intra-abdominal infections | No | Alandijany 2023^41^ |
| Bictegravir | Integrase Strand Transfer Inhibitor | In combination with other drugs for the treatment of Human immunodeficiency virus | No | Li 2023^27^ |
| cabotegravir | Integrase Strand Transfer Inhibitor | Prophylaxis Human immunodeficiency virus | No | Li 2023,^27^ Patel 2023^42^ |
| Dolutegravir | Integrase Strand Transfer Inhibitor | In combination with other drugs for the treatment of Human immunodeficiency virus | No | Li 2023,^27^ Lythgoe 2024,^43^ Sahoo 2023^22^ |
| Elvitegravir | Integrase Strand Transfer Inhibitor | Human immunodeficiency virus | No | Patel 2023^42^ |
| Baricitinib | Janus kinase (JAK) inhibitor | Rheumatoid arthritis, Atopic dermatitis, Alopecia areata, Juvenile idiopathic arthritis | No | Rabaan 2024^26^ |
| Capmatinib | MET tyrosine kinase inhibitor | Non-small cell lung cancer | Yes | Li 2023,^27^ Srivastava 2024^44^ |
| Tepotinib | MET tyrosine kinase inhibitor | Non-small cell lung cancer | No | Abduljalil 2023,^41^ Li 2023^27^ |
| Tipranavir | Non-peptidic protease inhibitor | Human immunodeficiency virus | No | Lythgoe 2024,^43^ Patel 2023,^42^ Sahoo 2023^22^ |
| Ribavirin | Nucleoside antiviral (RNA synthesis inhibitor) | In combination with other drugs for chronic hepatitis C | Yes | Ezat 2023,^32^ Hashemi 2024^25^ |
| Zidovudine | Nucleoside reverse transcriptase inhibitor | Human immunodeficiency virus | Yes | Bhattacharjee 2024,^29^ Patel 2023,^42^ Rabaan 2024^26^ |
| Naldemedine | Opioid antagonist | Opioid-induced constipation | Yes | Srivastava 2024^44^ |
| Cannabidiol | Phytocannabinoid | Seizures associated with tuberous sclerosis complex, Lennox-Gastaut syndrome, Multiple sclerosis | No | Paul 2024^45^ |
| Amphotericin | Polyene antifungal | Mycoses, Visceral leishmaniasis, Fungal infections, Candidiasis | No | Peruzzu 2023^46^ |
| Mefloquine hydrochloride | Quinoline antimalarial | P. falciparum malaria | No | Horton 2025^24^ |
| Cefiderocol | Siderophore cephalosporin antibiotic | Gram-negative infections | No | Sahoo 2023^22^ |
| Fostamatinib | Spleen tyrosine kinase inhibitor | Chronic immune thrombocytopenia | No | Paul 2024,^45^ Saha 2024^47^ |
| Doxycycline | Tetracycline antibiotic | Respiratory tract infections, Urinary tract infections, Sexually transmitted infections, Skin infections | No | Yousaf 2025^37^ |
| Adalimumab | Tumour necrosis factor (TNF) inhibitor | Ankylosing spondylitis, Psoriatic arthritis, Hidradenitis suppurativa, Crohn's disease | No | Rabaan 2024^26^ |
| Etanercept | Tumour necrosis factor (TNF) inhibitor | Psoriatic arthritis, Axial spondyloarthritis, Plaque psoriasis | No | Rabaan 2024^26^ |
| Infliximab | Tumour necrosis factor (TNF) inhibitor | Crohn's disease, Ulcerative colitis, Ankylosing spondylitis, Psoriatic arthritis, Psoriasis | Yes | Rabaan 2024^26^ |
| Imatinib | Tyrosine kinase inhibitor | Chronic myelogenous leukemia, Acute lymphoblastic leukemia, Myelodysplastic/myeloproliferative diseases | No | Dutt 2023,^39^ Ezat 2023,^32^ Rabaan 2024^26^ |
| Nilotinib | Tyrosine kinase inhibitor | Chronic myelogenous leukemia | No | Khan 2024,^40^ Li 2023^27^ |
| Ponatinib | Tyrosine kinase inhibitor | Chronic myeloid leukaemia, Acute lymphoblastic leukaemia | Yes | Khan 2024^40^ |

**Supplementary Table 5 – Potential candidates for the treatment of MERS-CoV, SARS-CoV, SARS-CoV-2 (COVID-19)**

| **Repurposed indication** | **Medicines** | **Therapeutic Class (A-Z)** | **UK/EU Approved Indications** | **Cancer Y/N** | **References** |
| --- | --- | --- | --- | --- | --- |
| COVID | Pyridostigmine bromide | Acetylcholinesterase inhibitor | Myasthenia gravis, Paralytic ileus, post-operative urinary retention | No | Jaimes-Castelan 2024^48^ |
| COVID | Disulfiram | Aldehyde dehydrogenase inhibitor | Alcohol abuse | No | Boulon 2024,^49^ Mia 2024,^50^ Nazir 2024^51^ |
| COVID | Busulfan | Alkylating agent | Chronic myeloid leukaemia, Polycythaemia vera, Thrombocythemia, Myelofibrosis | Yes | Alzahrani 2024^52^ |
| COVID | Carboplatin | Alkylating agent | Ovarian carcinoma of epithelial origin, small cell lung carcinoma | Yes | Alzahrani 2024^52^ |
| COVID | Cisplatin | Alkylating agent | Testicular cancer, Bladder cancer, Ovarian cancer, Head and neck cancer, non-small cell lung cancer, small cell lung cancer | Yes | Brun 2025^53^ |
| COVID | Ifosfamide | Alkylating agent | Malignant disease | Yes | Alzahrani 2024^52^ |
| COVID | Dexmedetomidine | Alpha-2 receptor agonist | Sedation in non-intubated patients | No | Kumawat 2024^54^ |
| COVID | Telmisartan | Angiotensin II receptor blocker | Hypertension, Cardiovascular prevention | No | Aviles-Alia 2024^55^ |
| COVID | Fosinopril | Angiotensin-Converting Enzyme inhibitor | Hypertension, Heart failure | No | Metwaly 2024^56^ |
| COVID | Glycopyrronium + formoterol fumarate | Anticholinergic (long-acting muscarinic antagonist) + long-acting beta-2 agonist | Chronic obstructive pulmonary disease | No | Enyeji 2024^57^ |
| COVID | Enoxaparin | Anticoagulant | Venous thromboembolic disease, Deep vein thrombosis, Prevention of thrombus formation, Myocardial infarction | No | Jaimes-Castelan 2024^48^ |
| COVID | Valproic acid | Antiepileptic | Epilepsy | No | Alzahrani 2024^52^ |
| COVID | Acrivastine | Antihistamine | Rhinitis, Urticaria | No | Zhong 2024^58^ |
| COVID | Azelastine | Antihistamine | Seasonal allergic conjunctivitis, Seasonal allergic rhinitis | No | Zhong 2024^58^ |
| COVID | Bilastine | Antihistamine | Allergic rhino-conjunctivitis, Urticaria. | No | Hamdan 2024^59^ |
| COVID | Desloratadine | Antihistamine | Allergic rhinitis, Urticaria | No | Zhong 2024^58^ |
| COVID | Diphenhydramine | Antihistamine | Allergic conditions of the skin | No | Zhong 2024^58^ |
| COVID | Fexofenadine | Antihistamine | Allergic rhinitis, Urticaria | No | Hamdan 2024^59^ |
| COVID | Loratadine | Antihistamine | Allergic rhinitis, Urticaria | No | Zhong 2024^58^ |
| COVID | Promethazine | Antihistamine | Allergic rhinitis, Urticaria, Anaphylactic reactions to drugs/ foreign proteins, Antiemetic, Insomnia and paediatric sedation, | No | Zeidan 2024^2^ |
| COVID | Rupatadine | Antihistamine | Allergic rhinitis, Urticaria | No | Hamdan 2024^59^ |
| COVID | Triprolidine | Antihistamine | In combination with other drugs for: Upper respiratory tract disorders | No | Zhong 2024^58^ |
| COVID | Chloroquine diphosphate | Antimalarial | Active rheumatoid arthritis, Malaria, Amoebic hepatitis and abscess, Discoid and systemic lupus erythematosus | No | Chavda 2024,^60^ Enyeji 2024,^57^ Islam 2024,^61^ Kumawat 2024,^54^ Mia 2024^50^ |
| COVID | Pyronaridine + Artesunate | Antimalarial | Malaria | No | Abla 2024^62^ |
| COVID | Hydroxychloroquine | Antimalarial and immunomodulatory agent | Rheumatoid arthritis, Discoid and systemic lupus erythematosus, Dermatological conditions, Lupus erythematosus | No | Chavda 2024,^60^ Enyeji 2024,^57^ Jaimes-Castelan 2024,^48^ Kumawat 2024,^54^ Martins 2024,^63^ Mia 2024,^50^ Singh 2024,^64^ Islam 2024^61^ |
| COVID | Rifampicin | Antimicrobial | Tuberculosis, leprosy, Brucellosis, Legionnaires Disease, Staphylococcal infections, Meningococcal meningitis, Haemophilus influenzae | No | Chakraborty 2024^65^ |
| COVID, MERS, SARS | Ivermectin | Antiparasitic | Anguillulosis, Microfilaraemia, Scabies, Inflammatory lesions of rosacea | No | Chavda 2024,^60^ Enyeji 2024,^57^ Gossen 2024,^66^ Jaimes-Castelan 2024,^48^ Mawazi 2024,^67^ Mia 2024,^50^ Pereira 2024,^68^ Singh 2024,^64^ Xu 2024^69^ |
| COVID | Pyrimethamine | Antiprotozoal | In combination with a synergistic agent for: Toxoplasmosis | No | Godde 2024^70^ |
| COVID, MERS, SARS | Chlorpromazine | Antipsychotic | Schizophrenia and other psychoses, Mania and hypomania, Anxiety, Psychomotor agitation, Excitement, Violent or dangerously impulsive behaviour, Intractable hiccup, Nausea and vomiting in terminal illness, Induction of hypothermia, Childhood schizophrenia, Autism | No | Zeidan 2024,^2^ Barghash 2024^4^ |
| COVID | Haloperidol | Antipsychotic | Schizophrenia and schizoaffective disorder, Delirium, Bipolar, Acute psychomotor agitation, Persistent aggression and psychotic symptoms, Tourette's syndrome | No | Boulon 2024,^49^ Zeidan 2024^2^ |
| COVID, MERS, SARS | Oseltamivir | Antiviral (Neuraminidase inhibitor) | Influenza | No | Gao 2024,^71^ Jaimes-Castelan 2024,^48^ Marinho 2025,^72^ Mia 2024,^50^ Waseem 2024,^73^ Barghash 2025^4^ |
| COVID, MERS, SARS | Zanamivir | Antiviral (Neuraminidase inhibitor) | Influenza A and B | No | Gao 2024,^71^ Mia 2024,^50^ Waseem 2024,^73^ Barghash 2024^4^ |
| COVID | Zanamivir | Antiviral (Neuraminidase inhibitor) | Influenza A and B | No | Gao 2024,^71^ Mia 2024,^50^ Waseem 2024,(71) Barghash 2024^4^ |
| COVID | Elbasvir | Antiviral (NS5A inhibitor) | In combination with other drugs for: Chronic hepatitis C | No | Ahmad 2024^74^ |
| COVID | Ledipasvir | Antiviral (NS5A inhibitor) | Chronic hepatitis C | No | Gao 2024,^71^ Pereira 2024^68^ |
| COVID | Sofosbuvir | Antiviral (NS5B polymerase inhibitor) | In combination with other drugs for: Chronic hepatitis C | No | Gao 2024,^71^ Kumawat 2024,^54^ Metwaly 2024,^56^ Singh 2024^64^ |
| COVID, MERS, SARS | Amantadine | Antiviral and antiparkinsonian | Parkinson's disease | No | Barghash 2024^4^ |
| COVID | Zepatier (Elbasvir/Grazoprevir) | Antivirals (NS5A inhibitor + NS3/4A protease inhibitor) | Fixed dose combination for: Chronic hepatitis C | No | Ahmad 2024^74^, Pereira 2024^68^ |
| COVID | Epclusa | Antivirals (NS5B polymerase inhibitor + NS5A inhibitor) | Licensed combination= sofosbuvir/velpatasvir for: chronic hepatitis C | No | Boulon 2024^49^ |
| COVID | Venetoclax | BCL-2 (B-cell lymphoma 2) inhibitor | Chronic lymphocytic leukaemia | Yes | Xu 2024^69^ |
| COVID, MERS, SARS | Metformin | Biguanide | Type 2 Diabetes, Polycystic ovary syndrome | No | Singh 2024,^64^ Yip 2025,^75^ Barghash 2024^4^ |
| COVID | Chenodeoxycholic acid | Bile acid | Cerebrotendinous xanthomatosis | No | Fiorucci 2024^76^ |
| COVID | Ursodeoxycholic acid | Bile acid | Cholesterol stones, Hepatobiliary disorder associated with cystic fibrosis | No | Fiorucci 2024,^76^ Lee 2024^77^ |
| COVID | Vemurafenib | BRAF kinase inhibitor | Melanoma | Yes | Alzahrani 2024,^52^ Bogacheva 2024^78^ |
| COVID | Diltiazem | Calcium channel blocker | Angina pectoris, Hypertension | No | Padey 2024^16^ |
| COVID | Digoxin | Cardiac glycoside | Cardiac failure, Supraventricular arrhythmias | No | Alzahrani 2024,^52^ Boulon 2024,^49^ Pereira 2024^68^ |
| COVID | Budesonide | Corticosteroid | Seasonal allergic rhinitis | No | Enyeji 2024,^57^ Martins 2024^63^ |
| COVID | Hydrocortisone | Corticosteroid | Adrenal insufficiency, Haemorrhoids, Pruritus ani, Eczema, Allergic contact dermatitis, Irritant contact dermatitis, Stings/bug bites, Photodermatitis, Otitis externa, Intertrigo, Prurigo nodularis, Seborrhoeic dermatitis, Congenital adrenal hyperplasia, Severe bronchial asthma, Drug hypersensitivity reactions | No | Alzahrani 2024^52^ |
| COVID | Aliskiren | Direct renin inhibitor | Essential hypertension | No | Metwaly 2024^56^ |
| COVID | Estradiol | Estrogen hormone | Hormone replacement therapy, Prevention of osteoporosis | No | Aviles-Alia 2024,^55^ Xu 2024^69^ |
| COVID | Ionafarnib | Farnesyltransferase Inhibitor | Hutchinson-Gilford progeroid syndrome, Processing-deficient progeroid laminopathies | No | Khan 2025^79^ |
| COVID | Ofloxacin | Fluoroquinolone antibiotic | Acute pyelonephritis and complicated urinary tract infections, non-gonococcal urethritis and cervicitis, Gonococcal urethritis and cervicitis due to susceptible Neisseria gonorrhoea’s, Acute exacerbations of chronic obstructive pulmonary disease, Uncomplicated cystitis, Urethritis | No | Chakraborty 2024^65^ |
| COVID | Miglustat | Glucosylceramide synthase inhibitor | Gaucher disease, Niemann-Pick type C disease, Pompe disease | No | Brun 2025^53^ |
| COVID | Riluzole | Glutamate release inhibitor | Amyotrophic lateral sclerosis | No | Marquez-Monino 2025^80^ |
| COVID | Teicoplanin | Glycopeptide antibiotic | Complicated skin and soft tissue infections, Bone and joint infections, Pneumonia, Urinary tract infections, Infective endocarditis, Peritonitis, Clostridium difficile infection | No | Espano 2024,^6^ Mia 2024,^50^ Barghash 2024^4^ |
| COVID | Simvastatin | HMG-CoA reductase inhibitor | Hypercholesterolaemia, Mixed dyslipidaemia, Cardiovascular prevention | No | Alzahrani 2024^52^ |
| COVID | Imiquimod | Immune response modifier | External genital and perianal warts, Superficial basal cell carcinomas, Actinic keratoses on face or scalp | No | Voloudakis 2025,^81^ Waseem 2024^73^ |
| COVID | Azathioprine | Immunosuppressant | Inflammatory bowel disease, Severe active rheumatoid arthritis, Systemic lupus erythematosus, Dermatomyositis, polymyositis, Auto-immune chronic active hepatitis, Pemphigus vulgaris, Polyarteritis nodosa, Auto-immune haemolytic anaemia, Chronic refractory idiopathic thrombocytopenic purpura | No | Alzahrani 2024,^52^ Voloudakis 2025^81^ |
| COVID | Ciclesonide | Inhaled corticosteroid | Asthma | No | Enyeji 2024^57^ |
| COVID | Dolutegravir | Integrase Strand Transfer Inhibitor | In combination with other drugs for: Human Immunodeficiency Virus | No | Kasgari 2025^82^ |
| COVID | Raltegravir | Integrase Strand Transfer Inhibitor | In combination with other drugs for: Human Immunodeficiency Virus | No | Gao 2024,^71^ Mohamed 2025^83^ |
| COVID | Anakinra | Interleukin-1 receptor antagonist | Rheumatoid arthritis, Periodic fever syndromes, Still's disease | No | Chavda 2024,^60^ Jaimes-Castelan 2024,^48^ Mawazi 2024^67^ |
| COVID | Baricitinib | Janus kinase (JAK) inhibitor | Rheumatoid arthritis, Atopic dermatitis, Alopecia areata, Juvenile idiopathic arthritis | No | Chavda 2024,^60^ Jaimes-Castelan 2024,^48^ Low 2025,^84^ Rahmani 2024^85^ |
| COVID | Ruxolitinib | Janus kinase (JAK) inhibitor | Myelofibrosis, Polycythaemia vera, Graft versus host disease | No | Chavda 2024^60^ |
| COVID | Tofacitinib | Janus kinase (JAK) inhibitor | Active polyarticular juvenile idiopathic arthritis, Juvenile psoriatic arthritis | No | Jaimes-Castelan 2024^48^ |
| COVID | Montelukast | Leukotriene receptor antagonist | Asthma, Allergic rhinitis associated with asthma | No | Hamdan 2024^59^ |
| COVID | Azithromycin | Macrolide antibiotic | Bacterial sinusitis, Bacterial otitis media, Pharyngitis, Tonsillitis, Chronic bronchitis, Community acquired pneumonia, Skin and soft tissue infections, uncomplicated Chlamydia trachomatis urethritis and cervicitis, conjunctivitis (eye drops), pelvic inflammatory disease, sinusitis | No | Chavda 2024,^60^ Enyeji 2024,^57^ Mia 2024,^50^ Paroczai 2024,^86^ Singh 2024^64^ |
| COVID | Fidaxomicin | Macrolide antibiotic | Clostridioides difficile infections | No | Protic 2024^87^ |
| COVID | Trametinib | MEK (mitogen-activated extracellular signal-regulated kinase) inhibitor | In combination with other drugs for: Melanoma, Non-small cell lung cancer | Yes | Alzahrani 2024^52^ |
| COVID | Sarilumab | Monoclonal antibody | Rheumatoid arthritis, Polymyalgia rheumatica | No | Chavda 2024^60^ |
| COVID | Siltuximab | Monoclonal antibody | Multicentric Castleman's disease | No | Chavda 2024^60^ |
| COVID | Tocilizumab | Monoclonal antibody | Rheumatoid arthritis, Active systemic juvenile idiopathic arthritis, Giant cell arteritis | No | Chavda 2024,^60^ Jaimes-Castelan 2024,^48^ Kumawat 2024,^54^ Okeowo 2024,^88^ Papp 2024,^89^ Low 2025,^84^ Rahmani 2024^85^ |
| COVID | Lithium | Mood stabilizer | Mania, Manic-depressive illness, Recurrent depression, Aggressive or self-mutilating behaviour, Bipolar | No | Zeidan 2024^2^ |
| COVID | Everolimus | mTOR (mechanistic Target of Rapamycin) inhibitor | Neuroendocrine tumours of pancreatic origin, Neuroendocrine tumours of gastrointestinal or lung origin, Renal cell carcinoma | Yes | Barghash 2024,^4^ Godde 2024,^70^ Ullah 2024,^90^ Voloudakis 2025,^81^ Xu 2024^69^ |
| COVID | Sirolimus | mTOR (mechanistic Target of Rapamycin) inhibitor | Organ rejection, Sporadic lymphangioleiomyomatosis | No | Bogacheva 2024,^78^ Godde 2024,^70^ Ullah 2024,^90^ Xu 2024^69^ |
| COVID | Dornase alfa | Mucolytic enzyme | Cystic fibrosis | No | Kumawat 2024^54^ |
| COVID | Aprepitant | Neurokinin-1 receptor antagonist | Prevent nausea and vomiting | No | Godde 2024^70^ |
| COVID | Timolol maleate | Non-selective beta-adrenergic blocker | Elevated intra-ocular pressure, Ocular hypertension, Secondary glaucoma | No | Alzahrani 2024^52^ |
| COVID | Celecoxib | Nonsteroidal anti-inflammatory drug (NSAID) | Osteoarthritis, Rheumatoid arthritis, Ankylosing spondylitis | No | Martins 2024^63^ |
| COVID | Naproxen | Nonsteroidal anti-inflammatory drug (NSAID) | Rheumatoid arthritis, Osteoarthrosis, Ankylosing spondylitis, Gout, Acute musculoskeletal disorders, Dysmenorrhoea | No | Martins 2024^63^ |
| COVID | Aspirin | Nonsteroidal anti-inflammatory drug (NSAID), antiplatelet | Analgesic, Antipyretic, Anti-inflammatory actions, Secondary prevention of thrombotic cerebrovascular or cardiovascular disease, Anti-thrombotic action | No | Jaimes-Castelan 2024^48^ |
| COVID, MERS, SARS | Ribavirin | Nucleoside antiviral (RNA synthesis inhibitor) | In combination with other drugs for: Chronic hepatitis C | No | Barghash 2024,^4^ Boulon 2024,^49^ Chan 2024,^91^ Chavda 2024,^60^ Jaimes-Castelan 2024,^48^ Kumawat 2024,^54^ Mia 2024^50^ |
| COVID | Zidovudine | Nucleoside reverse transcriptase inhibitor | Human Immunodeficiency Virus | No | Alzahrani 2024,^52^ Gao 2024^71^ |
| COVID | Tenofovir | Nucleotide reverse transcriptase inhibitor | Chronic hepatitis B | No | Gao 2024,^71^ Kumawat 2024,^54^ Singh 2024,^64^ Waseem 2024^73^ |
| COVID | Cobicistat | Pharmacokinetic enhancer | In combination with other drugs for: Human Immunodeficiency Virus | No | Gallucci 2024,^92^ Pereira 2024^68^ |
| COVID, MERS, SARS | Cobicistat/darunavir | Pharmacokinetic enhancer + protease inhibitor | In combination with other drugs for: Human Immunodeficiency Virus | No | Barghash 2024^4^ |
| COVID | Duvelisib | Phosphoinositide 3-kinase inhibitor | Chronic lymphocytic leukaemia, Follicular lymphoma | Yes | Bogacheva 2024^78^ |
| COVID | Temoporfin | Photosensitizer | Head and neck squamous cell carcinoma | Yes | Mendonca 2024^93^ |
| COVID | Verteporfin | Photosensitizer | Exudative age-related macular degeneration, Subfoveal choroidal neovascularisation secondary to pathological myopia | No | Mendonca 2024^93^ |
| COVID | Cannabidiol | Phytocannabinoid | Seizures associated with tuberous sclerosis complex, Lennox-Gastaut syndrome, Multiple sclerosis | No | Nazir 2024^51^ |
| COVID | Rucaparib | Poly (ADP-ribose) polymerase (PARP) inhibitor | Epithelial ovarian, Fallopian tube, or Primary peritoneal cancer | Yes | Papp 2024^89^ |
| COVID | Amiloride | Potassium-sparing diuretic | Oedema, Hypertension | No | Brun 2025^53^ |
| COVID | Aprotinin | Protease inhibitor | Reduce blood loss, Blood transfusion | No | Padin 2024^94^ |
| COVID | Atazanavir | Protease inhibitor | In combination with ritonavir for: Human Immunodeficiency Virus | No | Metwaly 2024^56^ |
| COVID | Darunavir | Protease inhibitor | Human Immunodeficiency Virus | No | Chavda 2024,^60^ Jaimes-Castelan 2024,^48^ Marinho 2025,^72^ Martins 2024,^63^ Metwaly 2024,^56^ Paroczai 2024,^86^ Pereira 2024^68^ |
| COVID | Lopinavir | Protease inhibitor | In combination with ritonavir (fixed dose combination) for: Human Immunodeficiency Virus | No | Chakraborty 2024,^65^ Chavda 2024,^60^ Enyeji 2024,^57^ Handa 2024,^95^ Hongyu 2024,^96^ Jaimes-Castelan 2024,^48^ Marinho 2025,^72^ Mawazi 2024,^67^ Metwaly 2024,^56^ Mia 2024,^50^ Paroczai 2024,^86^ Singh 2024,^64^ Barghash 2024^4^ |
| COVID | Lopinavir/Ritonavir | Protease inhibitor | Human Immunodeficiency Virus | No | Enyeji 2024,^57^ Hongyu 2024,^96^ Mawazi 2024,^67^ Mia 2024^50^ |
| COVID, MERS, SARS | Lopinavir/Ritonavir + ribavirin | Protease inhibitor + Nucleoside antiviral (RNA synthesis inhibitor) | Human Immunodeficiency Virus \| In combination with other medicinal products for: Chronic hepatitis C | No | Enyeji 2024,^57^ Hongyu 2024,^96^ Mawazi 2024,^67^ Mia 2025^50^ |
| COVID | Carfilzomib | Proteasome inhibitor | In combination with other drugs for: Multiple myeloma | Yes | Metwaly 2024,^56^ Low 2025,^84^ Rahmani 2024^85^ |
| COVID | Fluoxetine | Selective serotonin reuptake inhibitor | Major depressive disorders, Obsessive-compulsive disorder, Bulimia nervosa | No | Kumawat 2024^54^ |
| COVID | Fluvoxamine | Selective serotonin reuptake inhibitor | Major depressive episode, obsessive compulsive disorder | No | Godde 2024,^70^ Jaimes-Castelan 2024,^48^ Prasanth 2024,^97^ Singh 2024,^64^ Wannigama 2024^98^ |
| COVID | Cefiderocol | Siderophore cephalosporin antibiotic | Gram-negative Infections | No | Ahmad 2024^74^ |
| COVID | Fostamatinib | Spleen tyrosine kinase inhibitor | Chronic immune thrombocytopenia | No | Bakshi 2025^99^ |
| COVID | Gliclazide | Sulfonylurea | Non-insulin-dependent diabetes | No | Yip 2025^75^ |
| COVID | Dexamethasone | Systemic corticosteroid | Non-infectious inflammatory conditions affecting the anterior segment of the eye, Cerebral oedema, Asthma, Skin diseases, Systemic lupus erythematodes, Systemic vasculitides, Rheumatoid arthritis, Still's disease, Idiopathic thrombocytopenic purpura, Tuberculous meningitis, Neoplastic diseases, Emesis induced by cytostatics, Emetogenic chemotherapy, Multiple myeloma, Acute lymphocytic leukaemia, Acute lymphoblastic leukaemia, General antiemetic treatment | No | Chavda 2024,^60^ Jaimes-Castelan 2024,^48^ Martins 2024,^63^ Okeowo 2024,^88^ Papp 2024,^89^ Paroczai 2024^86^ |
| COVID | Methylprednisolone | Systemic corticosteroid | Rheumatoid arthritis, Lupus erythematosus, Stevens-Johnson syndrome, Bronchial asthma, Drug hypersensitivity reactions, Angioneurotic oedema, Ulcerative colitis, Crohn's disease, Tuberculosis, Aspiration of gastric contents, Tuberculosis meningitis, Osteo-arthritis with an inflammatory component, Synovitis not associated with infection, Epicondylitis, Tenosynovitis, Plantar fasciitis, Bursitis, Keloids, Localized lichen planus, Localized lichen simplex, Granuloma annulare, Alopecia areata | No | Enyeji 2024,^57^ Jaimes-Castelan 2024,^48^ Martins 2024^63^ |
| COVID | Doxycycline | Tetracycline antibiotic | Respiratory tract infections, Urinary tract infections, sexually transmitted infections, Skin infections, Eye infections, Rickettsial infections, Rosacea, cholera, Bubonic plague, Louse and tick-borne relapsing fever, Tularaemia glanders, Melioidosis, Chloroquine-resistant falciparum malaria, Acute intestinal amoebiasis | No | Enyeji 2024^57^ |
| COVID | Minocycline | Tetracycline antibiotic | Acne | No | Chakraborty 2024^65^ |
| COVID | Etopophos | Topoisomerase II inhibitor | In combination with other drugs for: Testicular cancer, small cell lung cancer, Hodgkin's lymphoma, non-Hodgkin’s lymphoma, Acute myeloid leukaemia, Gestational trophoblastic neoplasia, Ovarian cancer | Yes | Alzahrani 2024^52^ |
| COVID | Entrectinib | Tyrosine kinase inhibitor | Solid tumours, non-small cell lung cancer | Yes | Ahmad 2024,^74^ Xu 2024^69^ |
| COVID | Imatinib | Tyrosine kinase inhibitor | Chronic myelogenous leukaemia, Acute lymphoblastic leukaemia, Myelodysplastic/myeloproliferative diseases, Hypereosinophilic syndrome, Gastrointestinal stromal tumours, Dermatofibrosarcoma protuberans | Yes | Ghavimehr 2024,^100^ Jaimes-Castelan 2024,^48^ Low 2025,^84^ Rahmani 2024^85^ |
| COVID | Nilotinib | Tyrosine kinase inhibitor | Chronic myelogenous leukaemia | Yes | Ghavimehr 2024,^100^ Malar 2024,^101^ Xu 2024^69^ |
| COVID | Vandetanib | Tyrosine kinase inhibitor | Thyroid cancer | Yes | Puhl 2023^102^ |
| COVID | Bevacizumab | Vascular Endothelial Growth Factor inhibitor | In combination with other drugs for: Carcinoma of the colon or rectum, Breast cancer, non-small cell lung cancer, Renal cell cancer, Epithelial ovarian, Fallopian tube, Primary peritoneal cancer, Cervical cancer | Yes | Jaimes-Castelan 2024^48^ |
| COVID | Tivozanib | Vascular Endothelial Growth Factor inhibitor | Renal cell carcinoma | Yes | Alzahrani 2024^52^ |
| COVID | Vincristine sulfate | Vinca alkaloid | Leukaemias, Lymphoma, Multiple myeloma, Solid tumours, Idiopathic thrombocytopenic purpura | Yes | Alzahrani 2024^52^ |
| COVID | Oseltamivir |  | Influenza | No | Gao 2024,^71^ Jaimes-Castelan 2024,^48^ Marinho 2025,^72^ Mia 2024,^50^ Waseem 2024,^73^ Barghash 2024^4^ |
| COVID | Ritonavir |  | Human Immunodeficiency Virus | No | Boulon 2024,^49^ Chavda 2024,^60^ Enyeji 2024,^57^ Gallucci 2024,^92^ Godde 2024,^70^ Hongyu 2024,^96^ Jaimes-Castelan 2024,^48^ Marinho 2025,^72^ Metwaly 2024,^56^ Mia 2024,^50^ Paroczai 2024,^86^ Singh 2024,^64^ Uzuner 2024^103^ |

**Supplementary Table 6 – Interventional clinical trials identified for influenza**

| **Medicines** | **Therapeutic Class** | **UK/EU Approved Indication** | **Cancer Y/N** | **Trial ID** | **Trial Phase** |
| --- | --- | --- | --- | --- | --- |
| Molnupiravir | Antiviral (RNA polymerase inhibitor) | COVID-19 | No | NCT05648448 ^104^ | Phase II |
| Dexamethasone sodium phosphate | Corticosteroid | Autoimmune disorders, rheumatology, oncology | No | NCT04366115 ^132^ | Phase I |
| Sirolimus + Oseltamivir | mTOR (mechanistic Target of Rapamycin) inhibitor + Antiviral (Neuraminidase inhibitor) | Sporadic lymphangioleiomyomatosis, organ rejection \| Influenza | No | NCT03901001 ^105^ | Phase III |
| N-acetylcysteine + Oseltamivir | Mucolytic agent + Antiviral (Neuraminidase inhibitor) | Respiratory tract diseases, Influenza | No | NCT03900988 ^106^ | Phase III |

**Supplementary Table 7 –** **Interventional clinical trials identified for SARS-CoV-2 (COVID-19)**

| **Medicines** | **Therapeutic Class (A-Z)** | **UK/EU Approved Indication** | **Cancer Y/N** | **Trial ID** | **Trial Phase** |
| --- | --- | --- | --- | --- | --- |
| Captopril | Angiotensin-Converting Enzyme (ACE) Inhibitors | Hypertension, Myocardial Infarction | No | NCT04345406 ^107^ | Phase III |
| Colchicine | Antigout agent | Acute gout | No | NCT04381936 ^108^ | Phase III |
| Hydroxychloroquine | Antimalarial and immunomodulatory agent | Rheumatoid arthritis, discoid and systemic lupus erythematosus | No | NCT05041907 ^109^ | Phase II |
| Hydroxychloroquine sulfate | Antimalarial and immunomodulatory agent | Rheumatoid arthritis, discoid and systemic lupus erythematosus | No | NCT05113810 ^110^ | Phase II |
| Hydroxychloroquine | Antimalarial and immunomodulatory agent | Rheumatoid arthritis, discoid and systemic lupus erythematosus | No | NCT04381936 ^108^ | Phase III |
| Hydroxychloroquine | Antimalarial and immunomodulatory agent | Rheumatoid arthritis, discoid and systemic lupus erythematosus | No | NCT04315948 ^111^ | Phase III |
| Hydroxychloroquine + Azithromycin | Antimalarial and immunomodulatory agent + Macrolide antibiotic | Rheumatoid arthritis, discoid and systemic lupus erythematosus\| Bacterial infections | No | NCT04334512 ^112^ | Phase II |
| Ivermectin | Antiparasitic | Intestinal strongyloidiasis, microfilaraemia, human sarcoptic scabies | No | NCT05041907 ^109^ | Phase II |
| Ivermectin | Antiparasitic | Intestinal strongyloidiasis, microfilaraemia, human sarcoptic scabies | No | NCT04351347 ^113^ | Phase II \|Phase III |
| Ivermectin | Antiparasitic | Intestinal strongyloidiasis, microfilaraemia, human sarcoptic scabies | No | NCT04703608 ^114^ | Phase III |
| Ivermectin | Antiparasitic | Intestinal strongyloidiasis, microfilaraemia, human sarcoptic scabies | No | NCT04885530 ^115^ | Phase III |
| Ivermectin | Antiparasitic | Intestinal strongyloidiasis, microfilaraemia, human sarcoptic scabies | No | NCT05040724 ^116^ | Phase III |
| Ivermectin | Antiparasitic | Intestinal strongyloidiasis, microfilaraemia, human sarcoptic scabies | No | NCT05736861 ^117^ | Phase III |
| Ivermectin | Antiparasitic | Intestinal strongyloidiasis, microfilaraemia, human sarcoptic scabies | No | NCT05894538 ^118^ | Phase III |
| Ivermectin + Doxycycline | Antiparasitic + Tetracycline antibiotic | Intestinal strongyloidiasis, microfilaraemia, human sarcoptic scabies\| Papulopustular lesions | No | NCT04482686 ^119^ | Phase I |
| Remdesivir + Baricitinib | Antiviral (RNA polymerase inhibitor) + Janus kinase (JAK) inhibitor | COVID-19 \| Rheumatoid arthritis, Atopic dermatitis, Alopecia areata, Juvenile idiopathic arthritis | No | NCT04321993 ^120^ | Phase II |
| Remdesivir + Dexamethasone + Apremilast | Antiviral (RNA polymerase inhibitor) + Corticosteroid + Phosphodiesterase inhibitors | COVID-19 \| Autoimmune disorders, rheumatology, oncology \| Psoriatic arthritis, psoriasis, oral ulcers | No | NCT04488081 ^121^ | Phase II |
| Remdesivir + Dexamethasone + Celecoxib + Famotidine | Antiviral (RNA polymerase inhibitor) + Corticosteroid + Nonsteroidal anti-inflammatory drug (NSAID) + Histamine H2-receptor antagonists | COVID-19 \| Autoimmune disorders, rheumatology, oncology \| Osteoarthritis, rheumatoid arthritis, ankylosing spondylitis \| Zollinger-Ellison syndrome, gastric ulcer, mild reflux oesophagitis | No | NCT04488081^121^ | Phase II |
| Remdesivir + Dexamethasone + ciclosporin | Antiviral (RNA polymerase inhibitor) + Corticosteroid + Immunosuppressants | COVID-19 \| Autoimmune disorders, rheumatology, oncology \| Endogenous uveitis, Rheumatoid arthritis, Psoriasis, Atopic dermatitis, Transplantation indications | No | NCT04488081^121^ | Phase II |
| Remdesivir + Dexamethasone + Dornase alfa | Antiviral (RNA polymerase inhibitor) + Corticosteroid + Recombinant human deoxyribonuclease I | COVID-19 \| Autoimmune disorders, rheumatology, oncology \| Cystic fibrosis | No | NCT04488081^121^ | Phase II |
| Remdesivir + Dexamethasone + Icatibant | Antiviral (RNA polymerase inhibitor) + Corticosteroid + Bradykinin B2 receptor antagonists | COVID-19 \| Autoimmune disorders, rheumatology, oncology \| Hereditary angioedema | No | NCT04488081^121^ | Phase II |
| Remdesivir + Imatinib Mesylate + Dexamethasone | Antiviral (RNA polymerase inhibitor) + Tyrosine-kinase inhibitors + Corticosteroid | COVID-19 \| Chronic myeloid leukaemia, gastrointestinal stromal tumours \| Autoimmune disorders, rheumatology, oncology | No | NCT04488081^121^ | Phase II |
| Amantadine Hydrochloride | Antiviral and antiparkinsonian | Parkinson's disease | No | NCT04854759 ^122^ | Phase III |
| Metformin | Biguanide | Type 2 diabetes mellitus | No | NCT05041907 ^109^ | Phase II |
| Metformin + Ivermectin | Biguanide + Antiparasitic | Type 2 diabetes mellitus \| Intestinal strongyloidiasis, microfilaraemia, human sarcoptic scabies | No | NCT04510194 ^123^ | Phase III |
| Metformin + Fluvoxamine | Biguanide + Selective Serotonin Reuptake Inhibitors (SSRIs) | Type 2 diabetes mellitus \| Major depressive episode, obsessive compulsive disorder | No | NCT04510194^123^ | Phase III |
| Budesonide | Corticosteroid | Seasonal allergic rhinitis | No | NCT04727424 ^124^ | Phase III |
| Fluticasone | Corticosteroid | Allergic rhinitis | No | NCT04885530 ^115^ | Phase III |
| Interferon beta 1a | Immunomodulator | Multiple sclerosis | No | NCT04518410 ^125^ | Phase II \|Phase III |
| Interferon beta-1b + Ribavirin | Immunomodulator + Nucleoside antiviral (RNA synthesis inhibitor) | Multiple sclerosis\| Chronic hepatitis C | No | NCT04494399 ^126^ | Phase II |
| Dimethyl fumarate | Immunomodulators | Multiple sclerosis | No | NCT04381936 ^108^ | Phase III |
| Anakinra | Interleukin-1 receptor antagonist | Rheumatoid Arthritis, Periodic fever syndromes, Still's Disease | No | NCT04381936^108^ | Phase III |
| Baricitinib | Janus kinase (JAK) inhibitor | Rheumatoid arthritis, Atopic dermatitis, Alopecia areata, Juvenile idiopathic arthritis | No | NCT04321993 ^120^ | Phase II |
| Baricitinib | Janus kinase (JAK) inhibitor | Rheumatoid arthritis, Atopic dermatitis, Alopecia areata, Juvenile idiopathic arthritis | No | NCT04381936 ^108^ | Phase III |
| Montelukast | Leukotriene receptor antagonist | Asthma | No | NCT04389411 ^127^ | Phase II \|Phase III |
| Montelukast | Leukotriene receptor antagonist | Asthma | No | NCT04885530 ^115^ | Phase III |
| Azithromycin | Macrolide antibiotic | Bacterial infections | No | NCT04381936 ^108^ | Phase III |
| Omalizumab | Monoclonal antibody | Allergic asthma, Chronic rhinosinusitis with nasal polyps | No | NCT04720612 ^128^ | Phase II |
| Tocilizumab | Monoclonal antibody | Rheumatoid arthritis, juvenile idiopathic arthritis, Giant Cell Arteritis | No | NCT04321993 ^120^ | Phase II |
| Aspirin | Nonsteroidal anti-inflammatory drug (NSAID), antiplatelet | Thrombotic cerebrovascular or cardiovascular disease, inflammatory conditions | No | NCT04381936 ^108^ | Phase III |
| Emtricitabine/Tenofovir Disoproxil Fumarate | Nucleoside Reverse Transcriptase Inhibitor (NRTI) | Human immunodeficiency virus (HIV-1) | No | NCT04890626 ^129^ | Phase III |
| Lopinavir/ritonavir | Protease inhibitor | Human immunodeficiency virus (HIV-1) | No | NCT05925140 ^130^ | Phase I |
| Lopinavir/ritonavir | Protease inhibitor | human immunodeficiency virus (HIV-1) | No | NCT04315948 ^111^ | Phase III |
| Lopinavir/ritonavir | Protease inhibitor | Human immunodeficiency virus (HIV-1) | No | NCT04381936 ^108^ | Phase III |
| Lopinavir/ritonavir + Interferon Beta-1A | Protease inhibitor + Immunomodulator | Human immunodeficiency virus (HIV-1) \| Multiple sclerosis | No | NCT04315948 ^111^ | Phase III |
| Fluoxetine | Selective serotonin reuptake inhibitor | Major depressive episodes, obsessive-compulsive disorder | No | NCT05041907 ^109^ | Phase II |
| Fluvoxamine | Selective serotonin reuptake inhibitor | Major depressive episodes, obsessive-compulsive disorder | No | NCT04885530^115^ | Phase III |
| Fluvoxamine | Selective serotonin reuptake inhibitor | Major depressive episode, obsessive compulsive disorder | No | NCT05894564 ^131^ | Phase III |
| Empagliflozin | Sodium-Glucose Co-Transporter 2 (SGLT2) Inhibitor | Type 2 diabetes mellitus, Heart failure, chronic kidney disease | No | NCT04381936 ^108^ | Phase III |
| Dexamethasone sodium phosphate | Systemic corticosteroid | Non-infectious inflammatory conditions affecting the anterior segment of the eye, Cerebral oedema, Asthma, Skin diseases, Systemic lupus erythematodes, Systemic vasculitides, Rheumatoid arthritis, Still's disease, Idiopathic thrombocytopenic purpura | No | NCT04366115^132^ | Phase I |
| Dexamethasone | Systemic corticosteroid | Non-infectious inflammatory conditions affecting the anterior segment of the eye, Cerebral oedema, Asthma, Skin diseases, Systemic lupus erythematodes, Systemic vasculitides, Rheumatoid arthritis, Still's disease, Idiopathic thrombocytopenic purpura | No | NCT04381936 ^108^ | Phase III |
| Infliximab | Tumour necrosis factor (TNF) inhibitor | Rheumatoid arthritis, Crohn's disease, Ulcerative colitis, Ankylosing spondylitis, Psoriatic arthritis, Psoriasis | No | NCT04330690 ^133^ | Phase III |
| Imatinib Mesylate | Tyrosine kinase inhibitor | Chronic myeloid leukaemia, gastrointestinal stromal tumours | Yes | NCT04488081 ^121^ | Phase II |
| Imatinib mesilate + Infliximab | Tyrosine kinase inhibitor + Tumour necrosis factor (TNF) inhibitor | Chronic myeloid leukaemia, gastrointestinal stromal tumours \| Rheumatoid arthritis, Crohn's disease, Ulcerative colitis, Ankylosing spondylitis, Psoriatic arthritis, Psoriasis | Yes | NCT05220280 ^134^ | Phase IV |

**References**

1 Almeida-Pinto F, Pinto R, Rocha J. Navigating the Complex Landscape of Ebola Infection Treatment: A Review of Emerging Pharmacological Approaches. *Infectious Diseases and Therapy*. 2024;13(1):21-55. Available from: <https://doi.org/10.1007/s40121-023-00913-y>.

2 Zeidan HA, Amer SK. Antiviral Potency of Antipsychotics: A New Therapeutic Strength. *International Journal of Pharmaceutical Quality Assurance*. 2024;15(4):2176-83. Available from: <https://doi.org/10.25258/ijpqa.15.4.12>.

3 Broni E, Ashley C, Adams J, Manu H, Aikins E, Okom M, et al. Cheminformatics-Based Study Identifies Potential Ebola VP40 Inhibitors. *International Journal of Molecular Sciences*. 2023;24(7):6298. Available from: <https://doi.org/10.3390/ijms24076298>.

4 Barghash RF, Gemmati D, Awad AM, Elbakry MMM, Tisato V, Awad K, et al. Navigating the COVID-19 Therapeutic Landscape: Unveiling Novel Perspectives on FDA-Approved Medications, Vaccination Targets, and Emerging Novel Strategies. *Molecules (Basel, Switzerland)*. 2024;29(23). Available from: <https://doi.org/10.3390/molecules29235564>.

5 Nascimento IJS, Santos-Junior PFS, de Araujo-Junior JX, da Silva-Junior EF. Strategies in Medicinal Chemistry to Discover New Hit Compounds against Ebola Virus: Challenges and Perspectives in Drug Discovery. *Mini-Reviews in Medicinal Chemistry*. 2022;22(22):2896-924. Available from: <https://doi.org/10.2174/1389557522666220404085858>.

6 Espano E, Kim J, Park SO, Padasas BT, Kim SH, Son JH, et al. Teicoplanin attenuates RNA virus infection in vitro. *bioRxiv*. 2024. Available from: <https://doi.org/10.1101/2024.09.29.615295>.

7 Kummer S, Lander A, Goretzko J, Kirchoff N, Rescher U, Schloer S. Pharmacologically induced endolysosomal cholesterol imbalance through clinically licensed drugs itraconazole and fluoxetine impairs Ebola virus infection in vitro. *Emerging Microbes and Infections*. 2022;11(1):195-207. Available from: <https://doi.org/10.1080/22221751.2021.2020598>.

8 Vanmechelen B, Stroobants J, Chiu W, Schepers J, Marchand A, Chaltin P, et al. Identification of novel Ebola virus inhibitors using biologically contained virus. *Antiviral Research*. 2022;200:105294. Available from: <https://doi.org/10.1016/j.antiviral.2022.105294>.

9 Martins KA, Wolfe DN. Marburg Virus Medical Countermeasures. *Methods in Molecular Biology*. 2025;2877:25-43. Available from: <https://doi.org/10.1007/978-1-0716-4256-6_2>.

10 Singh RK, Sarkar K, Das RK. In-silico repurposing of antiviral compounds against Marburg virus: a computational drug discovery approach. *In Silico Pharmacology*. 2025;13(1):41. Available from: <https://doi.org/10.1007/s40203-025-00323-7>.

11 Taye B, Thunauer R, Sugrue RJ, Maurer-Stroh S, Kosinski J. Identifying repurposed drugs with moderate anti-influenza virus activity through computational prioritization of drug-target pairs. *bioRxiv*. 2023. Available from: <https://doi.org/10.1101/2023.07.31.551116>.

12 Ghimire S, Sahukhal S, Shrestha A, Adhikari S, Subedi S, Budha KR, et al. REPURPOSING OF DRUGS AGAINST MUTATED STRAIN OF EURASIAN AVIAN LIKE H1N1 (EA H1N1) SWINE FLU VIRUS, GENOTYPE 4(G4) VIRUS. *bioRxiv*. 2022. Available from: <https://doi.org/10.1101/2022.10.20.512704>.

13 Bordoloi S, Prasad R, Lakshmi VS, Chandramohanadas R, Natarajan K, Nelson-Sathi S. Structure-based virtual screening and Molecular Dynamic Simulations identified FDA-approved molecules as potential inhibitors against the surface proteins of H1N1. *bioRxiv*. 2023. Available from: <https://doi.org/10.1101/2023.12.02.569695>.

14 Mtambo SE, Kumalo HM. In Silico Drug Repurposing of FDA-Approved Drugs Highlighting Promacta as a Potential Inhibitor of H7N9 Influenza Virus. *Molecules (Basel, Switzerland)*. 2022;27(14). Available from: <https://doi.org/10.3390/molecules27144515>.

15 Xie D, He S, Han L, Wu L, Huang H, Tao H, et al. Systematic optimization of host-directed therapeutic targets and preclinical validation of repositioned antiviral drugs. *Briefings in Bioinformatics*. 2022;23(3). Available from: <https://doi.org/10.1093/bib/bbac047>.

16 Padey B, Droillard C, Duliere V, Fouret J, de Lamballerie CN, Milesi C, et al. Host-Directed Repurposed Diltiazem Enhances the Antiviral Activity of Classic Antivirals Against Influenza and Sars-Cov-2. *SSRN*. 2024. Available from: <https://doi.org/10.2139/ssrn.5041998>.

17 Meineke R, Stelz S, Busch M, Werlein C, Kuhnel M, Jonigk D, et al. FDA-Approved Inhibitors of RTK/Raf Signaling Potently Impair Multiple Steps of In Vitro and Ex Vivo Influenza A Virus Infections. *Viruses*. 2022;14(9):2058. Available from: <https://doi.org/10.3390/v14092058>.

18 Sun Y, Wu J, Shen B, Yang H, Cui H, Han W, et al. Discovery of TRPV4-Targeting Small Molecules with Anti-Influenza Effects Through Machine Learning and Experimental Validation. *International Journal of Molecular Sciences*. 2025;26(3):1381. Available from: <https://doi.org/10.3390/ijms26031381>.

19 Li Y, Huo S, Yin Z, Tian Z, Huang F, Liu P, et al. The current state of research on influenza antiviral drug development: drugs in clinical trial and licensed drugs. *mBio*. 2023;14(5). Available from: <https://doi.org/10.1128/mbio.01273-23>.

20 Podduturi S, Vemula D, Singothu S, Bhandari V. In-silico investigation of E8 surface protein of the monkeypox virus to identify potential therapeutic agents. *Journal of Biomolecular Structure and Dynamics*. 2024;42(16):8242-55. Available from: <https://doi.org/10.1080/07391102.2023.2245041>.

21 Preet G, Oluwabusola ET, Milne BF, Ebel R, Jaspars M. Computational Repurposing of Mitoxantrone-Related Structures against Monkeypox Virus: A Molecular Docking and 3D Pharmacophore Study. *International Journal of Molecular Sciences*. 2022;23(22):14287. Available from: <https://doi.org/10.3390/ijms232214287>.

22 Sahoo AK, Augusthian PD, Muralitharan I, Vivek-Ananth RP, Kumar K, Kumar G, et al. In silico identification of potential inhibitors of vital monkeypox virus proteins from FDA approved drugs. *Molecular Diversity*. 2023;27(5):2169-84. Available from: <https://doi.org/10.1007/s11030-022-10550-1>.

23 Vuorio A, Raal F, Kovanen PT. Monkeypox is a global public health emergency: The role of repurposing cholesterol lowering drugs not to be forgotten. *Journal of Clinical Lipidology*. 2022;16(5):757-8. Available from: <https://doi.org/10.1016/j.jacl.2022.08.003>.

24 Horton A, Berryman H, Surani YM, Bewley K, Wand ME, Sutton JM, et al. The antiviral activity of licensed therapeutics against Mpox clade Ib, in vitro; alternative options for the treatment of Mpox. *bioRxiv*. 2025. Available from: <https://doi.org/10.1101/2025.01.11.632516>.

25 Hashemi M, Zabihian A, Hajsaeedi M, Hooshmand M. Antivirals for monkeypox virus: Proposing an effective machine/deep learning framework. *PloS One*. 2024;19(9 September):e0299342. Available from: <https://doi.org/10.1371/journal.pone.0299342>.

26 Rabaan AA, Alfaresi M, Alrasheed HA, Al Kaabi NA, Abduljabbar WA, Al Fares MA, et al. Network-Based Drug Repurposing and Genomic Analysis to Unveil Potential Therapeutics for Monkeypox Virus. *Chemistry and Biodiversity*. 2024;21(11):e202400895. Available from: <https://doi.org/10.1002/cbdv.202400895>.

27 Li V, Lee Y, Lee C, Kim H. Repurposing existing drugs for monkeypox: applications of virtual screening methods. *Genes and Genomics*. 2023;45(11):1347-55. Available from: <https://doi.org/10.1007/s13258-023-01449-8>.

28 Aldhaeefi M, Rungkitwattanakul D, Unonu J, Franklin CJ, Lyons J, Hager K, et al. The 2022 human monkeypox outbreak: Clinical review and management guidance. *American journal of health-system pharmacy : AJHP : official journal of the American Society of Health-System Pharmacists*. 2023;80(2):44-52. Available from: <https://doi.org/10.1093/ajhp/zxac300>.

29 Bhattacharjee A, Ahammad I, Chowdhury ZM, Das KC, Keya CA, Salimullah M. Proteome-Based Investigation Identified Potential Drug Repurposable Small Molecules Against Monkeypox Disease. *Molecular Biotechnology*. 2024;66(4):626-40. Available from: <https://doi.org/10.1007/s12033-022-00595-w>.

30 Bojkova D, Zoller N, Tietgen M, Steinhorst K, Bechtel M, Rothenburger T, et al. Repurposing of the antibiotic nitroxoline for the treatment of mpox. *Journal of Medical Virology*. 2023;95(3):e28652. Available from: <https://doi.org/10.1002/jmv.28652>.

31 Borkotoky S, Prakash A, Modi GP, Dubey VK. Computational repurposing of potential dimerization inhibitors against sars-cov-2 main protease. *Letters in Drug Design and Discovery*. 2024;21(4):799-808. Available from: <https://doi.org/10.2174/1570180820666230111141203>.

32 Ezat AA, Abduljalil JM, Elghareib AM, Samir A, Elfiky AA. The discovery of novel antivirals for the treatment of mpox: is drug repurposing the answer? *Expert Opinion on Drug Discovery*. 2023;18(5):551-61. Available from: <https://doi.org/10.1080/17460441.2023.2199980>.

33 Islam MR, Hossain MJ, Roy A, Hasan AHMN, Rahman MA, Shahriar M, et al. Repositioning potentials of smallpox vaccines and antiviral agents in monkeypox outbreak: A rapid review on comparative benefits and risks. *Health Science Reports*. 2022;5(5):e798. Available from: <https://doi.org/10.1002/hsr2.798>.

34 Lam HYI, Guan JS, Mu Y. In silico repurposed drugs against monkeypox virus. *bioRxiv*. 2022. Available from: <https://doi.org/10.1101/2022.07.17.500371>.

35 Rejinold NS, Jin GW, Choy JH. Harnessing Nanohybridized Niclosamide for Precision Mpox Therapeutics. *Advanced Healthcare Materials*. 2025;14(14):2404818. Available from: <https://doi.org/10.1002/adhm.202404818>.

36 Shannon A, Canard B. Nucleotide analogues and mpox: Repurposing the repurposable. *Antiviral Research*. 2025;234:106057. Available from: <https://doi.org/10.1016/j.antiviral.2024.106057>.

37 Yousaf MA, Michel M, Khan ATA, Noreen M, Bano S. Repurposing doxycycline for the inhibition of monkeypox virus DNA polymerase: a comprehensive computational study. *In Silico Pharmacology*. 2025;13(1):27. Available from: <https://doi.org/10.1007/s40203-025-00307-7>.

38 Witwit H, Cubitt B, Khafaji R, Castro EM, Goicoechea M, Lorenzo MM, et al. Repurposing Drugs for Synergistic Combination Therapies to Counteract Monkeypox Virus Tecovirimat Resistance. *Viruses*. 2025;17(1):92. Available from: <https://doi.org/10.3390/v17010092>.

39 Dutt M, Kumar A, Rout M, Dehury B, Martinez G, Ndishimye P, et al. Drug repurposing for Mpox: Discovery of small molecules as potential inhibitors against DNA-dependent RNA polymerase using molecular modeling approach. *Journal of Cellular Biochemistry*. 2023;124(5):701-15. Available from: <https://doi.org/10.1002/jcb.30397>.

40 Khan AAS, Yousaf MA, Azhar J, Maqbool MF, Bibi R. Repurposing FDA approved drugs against monkeypox virus DNA dependent RNA polymerase: virtual screening, normal mode analysis and molecular dynamics simulation studies. *VirusDisease*. 2024;35(2):260-70. Available from: <https://doi.org/10.1007/s13337-024-00869-8>.

41 Alandijany TA, El-Daly MM, Tolah AM, Bajrai LH, Khateb AM, Kumar GS, et al. A multi-targeted computational drug discovery approach for repurposing tetracyclines against monkeypox virus. *Scientific Reports*. 2023;13(1):14570. Available from: <https://doi.org/10.1038/s41598-023-41820-z>.

42 Patel CN, Mall R, Bensmail H. AI-driven drug repurposing and binding pose meta dynamics identifies novel targets for monkeypox virus. *Journal of Infection and Public Health*. 2023;16(5):799-807. Available from: <https://doi.org/10.1016/j.jiph.2023.03.007>.

43 Lythgoe MP, Emhardt AJ, Naci H, Krell J, Sullivan R, Aggarwal A. Efficacy and safety of interim oncology treatments introduced for solid cancers during the COVID-19 pandemic in England: a retrospective evidence-based analysis. *The Lancet Regional Health - Europe*. 2024;46:101062. Available from: <https://doi.org/10.1016/j.lanepe.2024.101062>.

44 Srivastava V, Naik B, Godara P, Das D, Mattaparthi VSK, Prusty D. Identification of FDA-approved drugs with triple targeting mode of action for the treatment of monkeypox: a high throughput virtual screening study. *Molecular Diversity*. 2024;28(3):1093-107. Available from: <https://doi.org/10.1007/s11030-023-10636-4>.

45 Paul D, Saha S, Basu S, Chakraborti T. Computational analysis of pathogen-host interactome for fast and low-risk in-silico drug repurposing in emerging viral threats like Mpox. *Scientific Reports*. 2024;14(1):18736. Available from: <https://doi.org/10.1038/s41598-024-69617-8>.

46 Peruzzu D, Fecchi K, Venturi G, Gagliardi MC. Repurposing Amphotericin B and Its Liposomal Formulation for the Treatment of Human Mpox. *International Journal of Molecular Sciences*. 2023;24(10):8896. Available from: <https://doi.org/10.3390/ijms24108896>.

47 Saha S, Chatterjee P, Nasipuri M, Basu S, Chakraborti T. Computational drug repurposing for viral infectious diseases: a case study on monkeypox. *Briefings in Functional Genomics*. 2024;23(5):570-8. Available from: <https://doi.org/10.1093/bfgp/elad058>.

48 Jaimes-Castelan EG, Gonzalez-Espinosa C, Magos-Guerrero GA, Arrieta-Cruz I, Jimenez-Estrada M, Reyes-Chilpa R, et al. Drugs and natural products for the treatment of COVID-19 during 2020, the first year of the pandemic. *Boletín Medico del Hospital Infantil de México*. 2024;81(1):53-72. Available from: <https://doi.org/10.24875/BMHIM.23000016>.

49 Boulon R, Mazeaud C, Farahani MD, Broquiere M, Iddir M, Charpentier T, et al. Repurposing Drugs and Synergistic Combinations as Potential Therapies for Inhibiting SARS-CoV-2 and Coronavirus Replication. *ACS Pharmacology and Translational Science*. 2024;7(12):4043-55. Available from: <https://doi.org/10.1021/acsptsci.4c00512>.

50 Mia ME, Howlader M, Akter F, Hossain MM. Preclinical and Clinical Investigations of Potential Drugs and Vaccines for COVID-19 Therapy: A Comprehensive Review With Recent Update. *Clinical Pathology*. 2024;17. Available from: <https://doi.org/10.1177/2632010X241263054>.

51 Nazir F, John Kombe Kombe A, Khalid Z, Bibi S, Zhang H, Wu S, et al. SARS-CoV-2 replication and drug discovery. *Molecular and Cellular Probes*. 2024;77:101973. Available from: <https://doi.org/10.1016/j.mcp.2024.101973>.

52 Alzahrani KJ. Repurposing of Anti-Cancer Drugs Against Moderate and Severe COVID Infection: A Network-Based Systems Biological Approach. *Nigerian Journal of Clinical Practice*. 2024;27(8):950-7. Available from: <https://doi.org/10.4103/njcp.njcp_873_23>.

53 Brun J, Arman BY, Hill ML, Kiappes JL, Alonzi DS, Makower LL, et al. Assessment of repurposed compounds against coronaviruses highlights the antiviral broad-spectrum activity of host-targeting iminosugars and confirms the activity of potent directly acting antivirals. *Antiviral Research*. 2025;237:106123. Available from: <https://doi.org/10.1016/j.antiviral.2025.106123>.

54 Kumawat P, Agarwal LK, Sharma K. An Overview of SARS-CoV-2 Potential Targets, Inhibitors, and Computational Insights to Enrich the Promising Treatment Strategies. *Current Microbiology*. 2024;81(7):169. Available from: <https://doi.org/10.1007/s00284-024-03671-3>.

55 Aviles-Alia AI, Zulaica J, Perez JJ, Rubio-Martinez J, Geller R, Granadino-Roldan JM. The Discovery of inhibitors of the SARS-CoV-2 S protein through computational drug repurposing. *Computers in Biology and Medicine*. 2024;171:108163. Available from: <https://doi.org/10.1016/j.compbiomed.2024.108163>.

56 Metwaly AM, Elkaeed EB, Alsfouk AA, Ibrahim IM, Elkady H, Eissa IH. Repurposing FDA-approved drugs for COVID-19: targeting the main protease through multi-phase in silico approach. *Antiviral Therapy*. 2024;29(6). Available from: <https://doi.org/10.1177/13596535241305536>.

57 Enyeji AM, Arora A, Mangat HS. Effective Treatment of COVID-19 Infection with Repurposed Drugs: Case Reports. *Viral Immunology*. 2024;37(6):298-307. Available from: <https://doi.org/10.1089/vim.2024.0034>.

58 Zhong G, Li J, Wang H. Identification of HRH1 as an alternative receptor for SARS-CoV-2: insights from viral inhibition by repurposable antihistamines. *mBio*. 2024;15(8). Available from: <https://doi.org/10.1128/mbio.01697-24>.

59 Hamdan M, Kulabas N, Kucukguzel I. In silico Evaluation of H1-Antihistamine as Potential Inhibitors of SARS-CoV-2 RNA-dependent RNA Polymerase: Repurposing Study of COVID-19 Therapy. *Turkish Journal of Pharmaceutical Sciences*. 2024;21(6):566-76. Available from: <https://doi.org/10.4274/tjps.galenos.2024.49768>.

60 Chavda V, Dodiya P, Apostolopoulos V. Adverse drug reactions associated with COVID-19 management. *Naunyn-Schmiedeberg's Archives of Pharmacology*. 2024;397(10):7353-76. Available from: <https://doi.org/10.1007/s00210-024-03137-0>.

61 Islam MA, Pathak K, Saikia R, Pramanik P, Das A, Talukdar P, et al. An in-depth analysis of COVID-19 treatment: Present situation and prospects. *Archiv der Pharmazie*. 2024;357(11):e2400307. Available from: <https://doi.org/10.1002/ardp.202400307>.

62 Abla N, Almond LM, Bonner JJ, Richardson N, Wells TNC, Mohrle JJ. PBPK-led assessment of antimalarial drugs as candidates for Covid-19: Simulating concentrations at the site of action to inform repurposing strategies. *Clinical and Translational Science*. 2024;17(7):e13865. Available from: <https://doi.org/10.1111/cts.13865>.

63 Martins GF, Castro TS, Ferreira DAC. Drug repurposing for the treatment of patients infected with SARS-CoV-2. *Network Modeling Analysis in Health Informatics and Bioinformatics*. 2024;13(1):15. Available from: <https://doi.org/10.1007/s13721-024-00453-6>.

64 Singh S, Boyd S, Schilling WHK, Watson JA, Mukaka M, White NJ. The relationship between viral clearance rates and disease progression in early symptomatic COVID-19: a systematic review and meta-regression analysis. *Journal of Antimicrobial Chemotherapy*. 2024;79(5):935-45. Available from: <https://doi.org/10.1093/jac/dkae045>.

65 Chakraborty A, Ghosh R, Soumya Mohapatra S, Barik S, Biswas A, Chowdhuri S. Repurposing of antimycobacterium drugs for COVID-19 treatment by targeting SARS CoV-2 main protease: An in-silico perspective. *Gene*. 2024;922:148553. Available from: <https://doi.org/10.1016/j.gene.2024.148553>.

66 Gossen KR, Zhang M, Nikolov ZL, Fernando SD, King MD. Binding behavior of receptor binding domain of the SARS-CoV-2 virus and ivermectin. *Scientific Reports*. 2024;14(1):2743. Available from: <https://doi.org/10.1038/s41598-024-53086-0>.

67 Mawazi SM, Fathima N, Mahmood S, Al-Mahmood SMA. Antiviral therapy for COVID-19 virus: A narrative review and bibliometric analysis. *American Journal of Emergency Medicine*. 2024;85:98-107. Available from: <https://doi.org/10.1016/j.ajem.2024.09.001>.

68 Pereira JA, Costa ED. PHYSICOCHEMICAL AND PHARMACOKINETIC ANALYSIS AND DOCKING OF DRUG REPOSITIONING AGAINST SARS-COV-2: AN IN SILICO STUDY. *Indian Drugs*. 2024;61(2):23-34. Available from: <https://doi.org/10.53879/id.61.02.14233>.

69 Xu J, Abdulsalam Khaleel R, Zaidan HK, Faisal Mutee A, Fahmi Fawy K, Gehlot A, et al. Discovery of common molecular signatures and drug repurposing for COVID-19/Asthma comorbidity: ACE2 and multi-partite networks. *Cell Cycle*. 2024;23(4):405-34. Available from: <https://doi.org/10.1080/15384101.2024.2340859>.

70 Godde NJ, O'Brien CM, Vincan E, Vashi A, Olliff S, Tran BM, et al. Use of Stem Cell-Derived Cardiomyocyte and Nasal Epithelium Models to Establish a Multi-Tissue Model Platform to Validate Repurposed Drugs Against SARS-CoV-2 Infection. *bioRxiv*. 2024. Available from: <https://doi.org/10.1101/2024.05.22.595397>.

71 Gao X, Wang C, Jiang Y, Zhang S, Zhang M, Liu L, et al. Evaluation of inhibition effect and interaction mechanism of antiviral drugs on main protease of novel coronavirus: Molecular docking and molecular dynamics studies. *Journal of Molecular Graphics and Modelling*. 2024;133:108873. Available from: <https://doi.org/10.1016/j.jmgm.2024.108873>.

72 Marinho AD, Braz HLB, de Moraes Silveira JA, Rocha DG, Jorge RJB, de Andrade GM. Lopinavir and Ritonavir have High Affinity Toward the SARS-CoV-2 S-protein Receptor-binding Domain Sequenced in Brazil. *BIO Integration*. 2025;6(1). Available from: <https://doi.org/10.15212/bioi-2024-0055>.

73 Waseem W, Zafar R, Jan MS, Alomar TS, Almasoud N, Rauf A, et al. Drug repurposing of FDA-approved anti-viral drugs via computational screening against novel 6M03 SARS-COVID-19. *Irish Journal of Medical Science*. 2024;193(1):73-83. Available from: <https://doi.org/10.1007/s11845-023-03473-9>.

74 Ahmad SS, Khalid M. Evaluations of FDA-approved Drugs Targeting 3CLP of SARS-CoV-2 Employing a Repurposing Strategy. *Combinatorial Chemistry and High Throughput Screening*. 2024;27(19):2805-15. Available from: <https://doi.org/10.2174/1386207325666220816125639>.

75 Yip JMX, Chiang GSH, Lee ICJ, Lehming-Teo R, Dai K, Dongol L, et al. Mitochondria and the Repurposing of Diabetes Drugs for Off-Label Health Benefits. *International Journal of Molecular Sciences*. 2025;26(1):364. Available from: <https://doi.org/10.3390/ijms26010364>.

76 Fiorucci S, Urbani G, Biagioli M, Sepe V, Distrutti E, Zampella A. Bile acids and bile acid activated receptors in the treatment of Covid-19. *Biochemical Pharmacology*. 2024;228:115983. Available from: <https://doi.org/10.1016/j.bcp.2023.115983>.

77 Lee K, Na Y, Kim M, Lee D, Choi J, Kim G, et al. Ursodeoxycholic acid may protect from severe acute respiratory syndrome coronavirus 2 Omicron variant by reducing angiotensin-converting enzyme 2. *Pharmacology Research and Perspectives*. 2024;12(2):e1194. Available from: <https://doi.org/10.1002/prp2.1194>.

78 Bogacheva MS, Kuivanen S, Potdar S, Hassinen A, Huuskonen S, Pohner I, et al. Drug repurposing platform for deciphering the druggable SARS-CoV-2 interactome. *Antiviral Research*. 2024;223:105813. Available from: <https://doi.org/10.1016/j.antiviral.2024.105813>.

79 Khan M, Irvin P, Park SB, Ivester HM, Ricardo-Lax I, Leek M, et al. Repurposing of lonafarnib as a treatment for SARS-CoV-2 infection. *JCI Insight*. 2025;10(1):e182704. Available from: <https://doi.org/10.1172/jci.insight.182704>.

80 Marquez-Monino MA, Santiveri CM, de Leon P, Camero S, Campos-Olivas R, Jimenez MA, et al. The ALS drug riluzole binds to the C-terminal domain of SARS-CoV-2 nucleocapsid protein and has antiviral activity. *Structure*. 2025;33(1):39-50.e6. Available from: <https://doi.org/10.1016/j.str.2024.10.025>.

81 Voloudakis G, Lee KM, Vicari JM, Zhang W, Hoagland D, Venkatesh S, et al. A genetically based computational drug repurposing framework for rapid identification of candidate compounds: application to COVID-19. *medRxiv*. 2025. Available from: <https://doi.org/10.1101/2025.01.10.25320348>.

82 Kasgari HA, Moradi S, Alikhani A, Ahmadian N. Effectiveness of dolutegravir in moderate severity COVID-19 patients: A single-center, randomized, double-blind, placebo-controlled trial. *BioImpacts*. 2025;15:29952. Available from: <https://doi.org/10.34172/bi.29952>.

83 Mohamed MA, Alanazi AF, Alanazi WA, Elsaman T, Mohamed MS, Eltayib EM. Repurposing of eluxadoline as a SARS-CoV-2 main protease inhibitor: E-Pharmacophore based virtual screening, molecular docking, MM-GBSA calculations, and molecular dynamics simulations studies. *Journal of Applied Pharmaceutical Science*. 2025;15(1):102-10. Available from: <https://doi.org/10.7324/JAPS.2024.204792>.

84 Low ZY, Wong KH, Yip AJW, Liew HL, Farouk IA, Lal SK, et al. Unravelling the Potential of Anticancer Drugs for SARS-CoV-2. *Current Pharmacology Reports*. 2025;11(1):9. Available from: <https://doi.org/10.1007/s40495-025-00390-6>.

85 Rahmani D, Jafari A, Kesharwani P, Sahebkar A. Molecular targets in SARS-CoV-2 infection: An update on repurposed drug candidates. *Pathology, Research and Practice*. 2024;263:155589. Available from: <https://doi.org/10.1016/j.prp.2024.155589>.

86 Paroczai D, Bikov A, Blidaru A, Bobu E, Lascu A, Mot CI, et al. Comparative efficacy of repurposed drugs lopinavir-ritonavir and darunavir-ritonavir in hospitalised COVID-19 patients: insights from a tertiary centre cohort. *Frontiers in Cellular and Infection Microbiology*. 2024;14:1496176. Available from: <https://doi.org/10.3389/fcimb.2024.1496176>.

87 Protic S, Crnoglavac Popovic M, Kalicanin N, Prodanovic O, Sencanski M, Milicevic J, et al. SARS-CoV-2 PLpro Inhibition: Evaluating in Silico Repurposed Fidaxomicin's Antiviral Activity Through In Vitro Assessment. *ChemistryOpen*. 2024;13(11):e202400091. Available from: <https://doi.org/10.1002/open.202400091>.

88 Okeowo OM, Olung NF, Ijomone OM, Adeagbo AS. Exploring Effective Therapeutic Approaches for COVID-19: A Review on Progress and Prospects. *Coronaviruses*. 2024;5(3):73-84. Available from: <https://doi.org/10.2174/0126667975267776231117052825>.

89 Papp H, Toth E, Bovari-Biri J, Banfai K, Juhasz P, Mahdi M, et al. The PARP inhibitor rucaparib blocks SARS-CoV-2 virus binding to cells and the immune reaction in models of COVID-19. *British Journal of Pharmacology*. 2024;181(23):4782-803. Available from: <https://doi.org/10.1111/bph.17305>.

90 Ullah S, Rahman W, Ullah F, Ullah A, Jehan R, Iqbal MN, et al. A molecular dynamics simulations analysis of repurposing drugs for COVID-19 using bioinformatics methods. *Journal of Biomolecular Structure and Dynamics*. 2024;42(18):9561-70. Available from: <https://doi.org/10.1080/07391102.2023.2256864>.

91 Chan JFW, Yuan S, Chu H, Sridhar S, Yuen KY. COVID-19 drug discovery and treatment options. *Nature Reviews Microbiology*. 2024;22(7):391-407. Available from: <https://doi.org/10.1038/s41579-024-01036-y>.

92 Gallucci L, Bazire J, Davidson AD, Shytaj IL. Broad-spectrum antiviral activity of two structurally analogous CYP3A inhibitors against pathogenic human coronaviruses in vitro. *Antiviral Research*. 2024;221:105766. Available from: <https://doi.org/10.1016/j.antiviral.2023.105766>.

93 Mendonca DA, Cadima-Couto I, Buga CC, Arnaut ZA, Schaberle FA, Arnaut LG, et al. Repurposing anti-cancer porphyrin derivative drugs to target SARS-CoV-2 envelope. *Biomedicine and Pharmacotherapy*. 2024;176:116768. Available from: <https://doi.org/10.1016/j.biopha.2024.116768>.

94 Padin JF, Perez-Ortiz JM, Redondo-Calvo FJ. Aprotinin (II): Inhalational Administration for the Treatment of COVID-19 and Other Viral Conditions. *International Journal of Molecular Sciences*. 2024;25(13):7209. Available from: <https://doi.org/10.3390/ijms25137209>.

95 Handa Y, Okuwaki K, Kawashima Y, Hatada R, Mochizuki Y, Komeiji Y, et al. Prediction of Binding Pose and Affinity of Nelfinavir, a SARS-CoV-2 Main Protease Repositioned Drug, by Combining Docking, Molecular Dynamics, and Fragment Molecular Orbital Calculations. *The journal of physical chemistry. B*. 2024;128(10):2249-65. Available from: <https://doi.org/10.1021/acs.jpcb.3c05564>.

96 Hongyu H, Wu T, He F, Chao M, Huang J, Wang X, et al. The binding mechanism of failed, in processing and succeed inhibitors target SARS-CoV-2 main protease. *Journal of Biomolecular Structure and Dynamics*. 2024;42(20):10565-76. Available from: <https://doi.org/10.1080/07391102.2023.2257800>.

97 Prasanth MI, Wannigama DL, Reiersen AM, Thitilertdecha P, Prasansuklab A, Tencomnao T, et al. A systematic review and meta-analysis, investigating dose and time of fluvoxamine treatment efficacy for COVID-19 clinical deterioration, death, and Long-COVID complications. *Scientific Reports*. 2024;14(1):13462. Available from: <https://doi.org/10.1038/s41598-024-64260-9>.

98 Wannigama DL, Hurst C, Phattharapornjaroen P, Hongsing P, Sirichumroonwit N, Chanpiwat K, et al. Early treatment with fluvoxamine, bromhexine, cyproheptadine, and niclosamide to prevent clinical deterioration in patients with symptomatic COVID-19: a randomized clinical trial. *eClinicalMedicine*. 2024;70:102517. Available from: <https://doi.org/10.1016/j.eclinm.2024.102517>.

99 Bakshi A, Gangopadhyay K, Basak S, De RK, Sengupta S, Dasgupta A. Integrating State-Space Modeling, Parameter Estimation, Deep Learning, and Docking Techniques in Drug Repurposing: A Case Study on COVID-19 Cytokine Storm. *Journal of the American Medical Informatics Association : JAMIA*. 2025. Available from: <https://doi.org/10.1093/jamia/ocaf035>.

100 Ghavimehr E, Zand A, SeyedAlinaghi SA, Karimi A, Arani HZ, Mirzapour P, et al. Safety and Efficacy of Imatinib, Nilotinib, and Artesunate in COVID-19 Patients: A Systematic Review of Current Evidence. *Anti-Infective Agents*. 2024;22(1):36-53. Available from: <https://doi.org/10.2174/2211352521666230714160740>.

101 Malar DS, Verma K, Prasanth MI, Tencomnao T, Brimson JM. Network analysis-guided drug repurposing strategies targeting LPAR receptor in the interplay of COVID, Alzheimer's, and diabetes. *Scientific Reports*. 2024;14(1):4328. Available from: <https://doi.org/10.1038/s41598-024-55013-9>.

102 Puhl AC, Lane TR, Ekins S. Learning from COVID-19: How drug hunters can prepare for the next pandemic. *Drug Discovery Today*. 2023;28(10):103723. Available from: <https://doi.org/10.1016/j.drudis.2023.103723>.

103 Uzuner U, Akkus E, Kocak A, Celik Uzuner S. Exploring epigenetic drugs as potential inhibitors of SARS-CoV-2 main protease: a docking and MD simulation study. *Journal of Biomolecular Structure and Dynamics*. 2024;42(13):6892-903. Available from: <https://doi.org/10.1080/07391102.2023.2236714>.

104 ClinicalTrials.gov. *A Phase 2 Trial Comparing Antiviral Treatments in Early Symptomatic Influenza (AD ASTRA)*. *Trial ID:* *NCT05648448*. 2022. Status: Recruiting. Available from: <https://clinicaltrials.gov/study/NCT05648448> [Accessed

105 ClinicalTrials.gov. *Adjunctive Sirolimus and Oseltamivir Versus Oseltamivir Alone for Treatment of Influenza*. *Trial ID:* *NCT03901001*. 2019. Status: Recruiting. Available from: <https://clinicaltrials.gov/study/NCT03901001> [Accessed

106 ClinicalTrials.gov. *Intravenous N-acetylcysteine and Oseltamivir Versus Oseltamivir in Adults Hospitalized With Influenza and Pneumonia*. *Trial ID:* *NCT03900988*. 2019. Status: Recruiting. Available from: <https://clinicaltrials.gov/study/NCT03900988> [Accessed

107 ClinicalTrials.gov. *Angiotensin Converting Enzyme Inhibitors in Treatment of Covid 19*. *Trial ID:* *NCT04345406*. 2020. Status: Not yet recruiting. Available from: <https://clinicaltrials.gov/study/NCT04345406> [Accessed

108 ClinicalTrials.gov. *Randomised Evaluation of COVID-19 Therapy (RECOVERY)*. *Trial ID:* *NCT04381936*. 2020. Status: Recruiting. Available from: <https://clinicaltrials.gov/study/NCT04381936> [Accessed

109 ClinicalTrials.gov. *Finding Treatments for COVID-19: A Trial of Antiviral Pharmacodynamics in Early Symptomatic COVID-19 (PLATCOV) (PLATCOV)*. *Trial ID:* *NCT05041907*. 2021. Status: Recruiting. Available from: <https://clinicaltrials.gov/study/NCT05041907> [Accessed

110 ClinicalTrials.gov. *The Potential Use of Nebulized Hydroxychloroquine for the Treatment of COVID-19*. *Trial ID:* *NCT05113810*. 2021. Status: Unknown status. Available from: <https://clinicaltrials.gov/study/NCT05113810> [Accessed

111 ClinicalTrials.gov. *Trial of Treatments for COVID-19 in Hospitalized Adults (DisCoVeRy)*. *Trial ID:* *NCT04315948*. 2020. Status: Completed. Available from: <https://clinicaltrials.gov/study/NCT04315948> [Accessed

112 ClinicalTrials.gov. *A Study of Quintuple Therapy to Treat COVID-19 Infection (HAZDpaC)*. *Trial ID:* *NCT04334512*. 2020. Status: Completed. Available from: <https://clinicaltrials.gov/study/NCT04334512> [Accessed

113 ClinicalTrials.gov. *The Efficacy of Ivermectin in Larger Doses in COVID-19 Treatment*. *Trial ID:* *NCT04351347*. 2020. Status: Recruiting. Available from: <https://clinicaltrials.gov/study/NCT04351347> [Accessed

114 ClinicalTrials.gov. *Prevention and Treatment for COVID -19 (Severe Acute Respiratory Syndrome Coronavirus 2 SARS-CoV-2) Associated Severe Pneumonia in the Gambia (PaTS-COVID)*. *Trial ID:* *NCT04703608*. 2021. Status: Unknown status. Available from: <https://clinicaltrials.gov/study/NCT04703608> [Accessed

115 ClinicalTrials.gov. *ACTIV-6: COVID-19 Study of Repurposed Medications*. *Trial ID:* *NCT04885530*. 2021. Status: Completed. Available from: <https://clinicaltrials.gov/study/NCT04885530> [Accessed

116 ClinicalTrials.gov. *Evaluation of the Impact of the Administration of Single Dose of Ivermectin in the Early Phase of COVID-19 (IVERCoV)*. *Trial ID:* *NCT05040724*. 2021. Status: Unknown status. Available from: <https://clinicaltrials.gov/study/NCT05040724> [Accessed

117 ClinicalTrials.gov. *ACTIV-6: COVID-19 Study of Repurposed Medications - Arm A (Ivermectin 400)*. *Trial ID:* *NCT05736861*. 2023. Status: Completed. Available from: <https://clinicaltrials.gov/study/NCT05736861> [Accessed

118 ClinicalTrials.gov. *ACTIV-6: COVID-19 Study of Repurposed Medications - Arm D (Ivermectin 600)*. *Trial ID:* *NCT05894538*. 2023. Status: Completed. Available from: <https://clinicaltrials.gov/study/NCT05894538> [Accessed

119 ClinicalTrials.gov. *Trial of Combination Therapy to Treat COVID-19 Infection*. *Trial ID:* *NCT04482686*. 2020. Status: Completed. Available from: <https://clinicaltrials.gov/study/NCT04482686> [Accessed

120 ClinicalTrials.gov. *Treatment of Moderate to Severe Coronavirus Disease (COVID-19) in Hospitalized Patients*. *Trial ID:* *NCT04321993*. 2020. Status: Active, not recruiting. Available from: <https://clinicaltrials.gov/study/NCT04321993> [Accessed

121 ClinicalTrials.gov. *I-SPY COVID-19 TRIAL: An Adaptive Platform Trial for Critically Ill Patients (I-SPY_COVID)*. *Trial ID:* *NCT04488081*. 2020. Status: Recruiting. Available from: <https://clinicaltrials.gov/study/NCT04488081> [Accessed

122 ClinicalTrials.gov. *The Use of Amantadine in the Prevention of Progression and Treatment of COVID-19 Symptoms (COV-PREVENT)*. *Trial ID:* *NCT04854759*. 2021. Status: Unknown status. Available from: <https://clinicaltrials.gov/study/NCT04854759> [Accessed

123 ClinicalTrials.gov. *COVID-OUT: Early Outpatient Treatment for SARS-CoV-2 Infection (COVID-19)*. *Trial ID:* *NCT04510194*. 2020. Status: Completed. Available from: <https://clinicaltrials.gov/study/NCT04510194> [Accessed

124 ClinicalTrials.gov. *Repurposed Approved and Under Development Therapies for Patients With Early-Onset COVID-19 and Mild Symptoms*. *Trial ID:* *NCT04727424*. 2021. Status: Recruiting. Available from: <https://clinicaltrials.gov/study/NCT04727424> [Accessed

125 ClinicalTrials.gov. *ACTIV-2: A Study for Outpatients With COVID-19*. *Trial ID:* *NCT04518410*. 2020. Status: Completed. Available from: <https://clinicaltrials.gov/study/NCT04518410> [Accessed

126 ClinicalTrials.gov. *IFN Beta-1b and Ribavirin for Covid-19*. *Trial ID:* *NCT04494399*. 2020. Status: Unknown status. Available from: <https://clinicaltrials.gov/study/NCT04494399> [Accessed

127 ClinicalTrials.gov. *The Covid-19 Outpatient Symptom Montelukast Oximetry Trial (COSMO)*. *Trial ID:* *NCT04389411*. 2020. Status: Unknown status. Available from: <https://clinicaltrials.gov/study/NCT04389411> [Accessed

128 ClinicalTrials.gov. *COVID-19 Immunologic Antiviral Therapy With Omalizumab (CIAO)*. *Trial ID:* *NCT04720612*. 2021. Status: Unknown status. Available from: <https://clinicaltrials.gov/study/NCT04720612> [Accessed

129 ClinicalTrials.gov. *Clinical Trial to Evaluate the Efficacy of Different Treatments in Patients With COVID-19*. *Trial ID:* *NCT04890626*. 2021. Status: Completed. Available from: <https://clinicaltrials.gov/study/NCT04890626> [Accessed

130 ClinicalTrials.gov. *LUSZ Treatment Efficacy in Hospitalized COVID-19 Patients (LUSZ_AVIST)*. *Trial ID:* *NCT05925140*. 2023. Status: Recruiting. Available from: <https://clinicaltrials.gov/study/NCT05925140> [Accessed

131 ClinicalTrials.gov. *ACTIV-6: COVID-19 Study of Repurposed Medications - Arm E (Fluvoxamine 100)*. *Trial ID:* *NCT05894564*. 2023. Status: Completed. Available from: <https://clinicaltrials.gov/study/NCT05894564> [Accessed

132 ClinicalTrials.gov. *Evaluating AVM0703 for Treatment of COVID-19 or Influenza-mediated ARDS (AVM0703)*. *Trial ID:* *NCT04366115*. 2020. Status: Not yet recruiting. Available from: <https://clinicaltrials.gov/study/NCT04366115> [Accessed

133 ClinicalTrials.gov. *Treatments for COVID-19: Canadian Arm of the SOLIDARITY Trial (CATCO)*. *Trial ID:* *NCT04330690*. 2020. Status: Unknown status. Available from: <https://clinicaltrials.gov/study/NCT04330690> [Accessed

134 ClinicalTrials.gov. *SOLIDARITY Finland Plus Long-COVID*. *Trial ID:* *NCT05220280*. 2022. Status: Recruiting. Available from: <https://clinicaltrials.gov/study/NCT05220280> [Accessed
